# Graphene Loading with Polypyrrole Nanoparticles for Trace-Level Detection of Ammonia at Room Temperature

**DOI:** 10.1021/acsami.1c10559

**Published:** 2021-08-19

**Authors:** Juan Casanova-Chafer, Polona Umek, Selene Acosta, Carla Bittencourt, Eduard Llobet

**Affiliations:** † Microsystems Nanotechnologies for Chemical Analysis (MINOS), Universitat Rovira i Virgili, 43007 Tarragona, Spain; ‡ Jožef Stefan Institute, 10000 Ljubljana, Slovenia; § Chimie des Interactions Plasma−Surface (ChIPS), Research Institute for Materials Science and Engineering, Université de Mons, 7000 Mons, Belgium

**Keywords:** graphene, polypyrrole nanoparticles, gas sensor, ammonia detection, room temperature, ambient monitoring

## Abstract

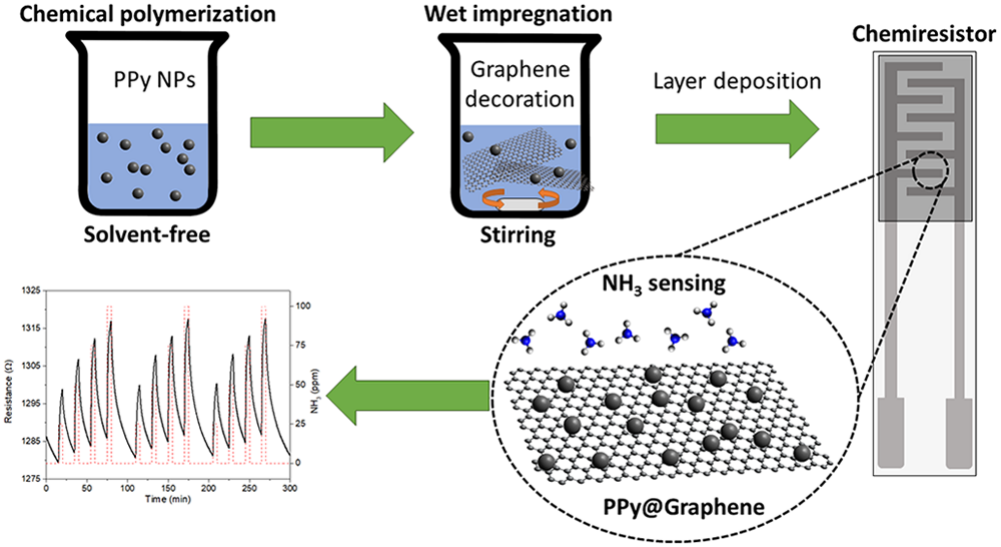

The outstanding versatility of graphene for surface functionalization has been exploited by its decoration with synthesized polypyrrole (PPy) nanoparticles (NPs). A green, facile, and easily scalable for mass production nanocomposite development was proposed, and the resulting PPy@Graphene was implemented in chemoresistive gas sensors able to detect trace levels of ammonia (NH_3_) under room-temperature conditions. Gas exposure for 5 min revealed that the presence of nanoparticles decorating graphene entail greater sensitivity (13-fold) in comparison to the bare graphene performance. Noteworthy, excellent repeatability (0.7% of relative error) and a low limit of detection of 491 ppb were obtained, together with excellent long-term stability. Besides, an extensive material characterization was conducted, and vibration bands obtained via Raman spectroscopy confirmed the formation of PPy NPs, while X-ray spectroscopy (XPS) revealed the relative abundance of the different species, as polarons and bipolarons. Additionally, XPS analyses were conducted before and after NH_3_ exposure to assess the PPy aging and the changes induced in their physicochemical and electronic properties. Specifically, the gas sensor was tested during a 5-month period, demonstrating significant stability over time, since just a slight decrease (11%) in the responses was registered. In summary, the present work reports for the first time the use of PPy NPs decorating graphene for gas-sensing purposes, revealing promising properties for the development of unattended gas-sensing networks for monitoring air quality.

## Introduction

During the lasts decades, air pollution has become a growing societal concern since it is associated with global climate change and shows noxious effects for both our environment and humankind.^1^ Exposure to gases at concentrations above the threshold limit values (TLV) or during long exposure times causes serious health issues. In fact, air pollution is linked to several million premature deaths per year.^2^ In consequence, governments and institutions worldwide are trying to overcome this problem that jeopardizes human health and environmental quality.

Natural sources of air pollution such as volcanic eruptions are not significant in comparison to those sources derived from human activity, such as industrial and combustion processes. However, since the replacement of the current energy sources toward renewable ones is progressive, real-time monitoring of the concentration of key pollutants is needed. With that, it is possible to reveal in space and time when the gases reach the TLV, and subsequently, different actions can be taken to reduce both air pollution and human exposure to those health-threatening levels.

Nevertheless, for achieving a ubiquitous, real-time monitoring of gases, developing a widespread sensor network possessing some requisite characteristics such as inexpensiveness, accuracy, durability, and low power consumption is needed.^3^ Inexpensive, yet reliable and accurate enough, sensor devices are key for achieving such gas-sensing networks.^4^ Some techniques, such as gas chromatography coupled to mass spectrometry (GC–MS), show significant drawbacks, as their complicated miniaturization and slow throughput complicate real-time air quality monitoring, despite being highly accurate.^5^ Other approaches, like the use of electrochemical sensors, present lower cost, but miniaturization and stability issues associated with electrolytes are still a challenge.^6^ For that reason, chemoresistive sensors have emerged as a promising option, as they gather many of the requirements needed in the next generation of inexpensive gas sensors.^7^

During the last decades, many research efforts have been focused on the development of chemoresistive sensors based on metal oxides (MOX). These devices usually show high sensitivity to gases and fast response and recovery dynamics, showing the potential for enabling ambient monitoring applications.^8^ However, some drawbacks are preventing their effective implementation in commercial devices. For instance, the selectivity of MOX-based sensors is still an issue, and usually, high operating temperatures are needed to activate the catalytic properties of MOX, leading to a high power consumption and lower durability due to the coalescence of MOX crystals into larger entities, altering their microstructure and, thus, their gas-sensing properties.^9^

In consequence, during the past few years, graphene has been attracting great interest as a gas-sensitive material due to its capability to work at room temperature.^10^ Indeed, graphene-based gas sensors are power lean and require simpler circuitry because there is no need to use heating elements. Besides, chemical vapor deposition of graphene is becoming a more mature nanotechnology (now can be produced in large quantities at relatively affordable cost) that shows superior properties, such as high carrier density and mobility, which is associated with low noise levels, and high surface area to volume ratio due to being a 2D nanomaterial.^11^ However, an important drawback of pure graphene-based gas sensors is their low sensitivity to gases. For that reason, further graphene modifications are often needed to improve its gas-sensing properties. The most common modifications consist of the direct functionalization of graphene (e.g., via wet chemistry, reactive plasma, or low-energy ion bombardment that generates defects and grafts functional groups) or its decoration with nanoparticles or nanocrystals (e.g., metals or metal oxides).^12,13^ However, the creation of a wide variety of graphene hybrids or composites is also feasible.^14^ With these approaches it is possible to obtain sensitive, inexpensive, and durable devices leveraging the high versatility of graphene to being surface functionalized.

To the best of our knowledge, some nanomaterials such as metal oxides have been widely used for detecting NH_3_. However, their use usually implies working temperatures of about a few hundred degrees Celsius.^15^ Conversely, fewer works are available for detecting NH_3_ using carbon-based nanomaterials at room temperature, probably because of their limited charge transfer and, in consequence, poor sensing responses.^16^ Indeed, carbonaceous nanomaterials like graphene and carbon nanotubes have been mainly focused on the detection of gases from combustion processes, such as nitrogen dioxide,^17^ with higher charge transfer than NH_3_, or even significantly hazardous ones such as benzene or hydrogen sulfide.^18^

Anthropogenic ammonia is related to industrial livestock production, crop agriculture, or fertilizer industries. According to the National Institute for Occupational Safety and Health (NIOSH), the permissible time weighted average (TWA) exposure limit is 25 ppm for 8 h, whereas the maximum short-term exposure limit (STEL) to NH_3_ is 35 ppm for 15 min.^19^ Not limited to this, the significant role of NH_3_ is well-known in additional environmental problems, such as promoting the formation of particulate matter (PM_2.5_).^20^ Therefore, there is a need for commercial gas-sensing devices able to selectively detect ammonia. In that way, the present paper combines two nanomaterials, graphene and polypyrrole, which have been demonstrated to show promise for detecting NH_3_ at room temperature.^21,22^

Functional conductive polymers, such as polypyrrole (PPy), polyaniline (PANI), and poly(3,4-ethylenedioxythiopene), probably assemble the requirements to be employed in the next generation of sensors. Their main advantages lie in their high sensitivity and their capability for room-temperature operation, large-scale production, and easy integration in wearable and flexible sensors.^23^ Specifically, polypyrrole has attracted far more research efforts than other polymers due to its superior properties such as biocompatibility and biodegradability, high conductivity, easy synthesis, and stability.^24,25^ In addition, the PPy morphology has a significant effect on the physicochemical and electronic properties due to the different arrangements found in PPy chains and their associated different charge distributions. In consequence, PPy has been synthesized in a wide range of nanostructures, such as nanoparticles,^26^ nanoribbons,^27^ and nanofibers,^28^ to cite some. These PPy forms are usually synthesized through electrochemical methods, in which the pyrrole monomers are electropolymerized in an electrolyte solution. The main advantage of this method is the high control over the geometry and thickness of the PPy synthesized by modulating the applied voltage and current density.^29^ However, its main drawbacks are associated with the relatively complex instrumentation and the difficulty of large-scale production.^30^

Thereby, it is worth noting that since polymers usually lead to sensitive gas detection of NH_3_ even under room-temperature conditions, the first nanocomposite of PPy NPs and graphene as a gas-sensitive layer presents a significant synergistic effect between the two nanomaterials. This novel approach shows outstanding gas-sensing performance, enabling a highly sensitive, reproducible, and stable NH_3_ detection, differing from the previously reported use of PPy films combined with different graphene configurations such as graphene oxide and reduced graphene oxide.^31,32^ Besides, the synthesis method proposed has been revealed as an easy, inexpensive, and scalable to large production approach. In addition, the nanomaterial deposition over the sensor electrodes through the airbrush method constitutes a straightforward and fast way that implies high throughputs. But not limited to this, the present work presents a green-chemistry approach in which low concentrations of NH_3_ are detected by using a solvent-free synthesis procedure and biocompatible nanomaterials.

## Experimental Section

### Synthesis of Polypyrrole Nanoparticles and Graphene Decoration

The synthesis of polypyrrole nanoparticles was conducted via chemical polymerization, modifying a protocol described in the literature.^33,34^ Poly(vinyl alcohol) (PVA) (Merck KGaA) was chosen as a water-soluble polymer to act as a dispersion aid. Since previous works already reported that greater molecular weights of PVA lead to smaller PPy nanoparticles,^33,35^ a PVA with *M*_w_ = 186 000 was chosen. First, a 7.5 wt % solution of PVA dissolved in distilled water was prepared under magnetic stirring at room temperature for 10 min. Afterward, 3.5 wt % iron chloride (FeCl_3_) (Merck KGaA) was added to the aqueous PVA solution, waiting 1 h to achieve the equilibration. In that time, the solution appearance changed from limpid to orange because FeCl_3_ acts as an oxidizing agent, forming the PVA/iron cation complex. Subsequently, the pyrrole monomer (C_4_H_5_N) (Merck KGaA) was added into the aqueous PVA/FeCl_3_ solution dropwise, with a molar ratio FeCl_3_/pyrrole of 2.5. Then, the solution was kept overnight under vigorous stirring to complete the polymerization. Meanwhile, the appearance changed from orange to black, indicating that polymerization took place derived from the contact of the pyrrole monomer with the iron cation. Nevertheless, the resulting nanoparticles should be extracted and cleaned via the centrifugation method from the solution. Specifically, three consecutive centrifugation processes (10 000 rpm and 10 min) were conducted, removing the dispersion solution and washing with abundant distilled water after each centrifugation. The resulting PPy NPs presented the form of a black powder that was dried in an oven at moderate temperature (80 °C).

Afterward, a graphene solution in distilled water (0.5 mg/mL) was prepared by using graphene nanoflakes (Strem Chemicals Inc.). Subsequently, to achieve a suitable graphene exfoliation, the solution was placed in an ultrasonic bath at high frequency (35 kHz) for 1 h. Then, 5% wt of PPy NPs was added to the graphene solution and immediately mixed under vigorous stirring for 2 h. This method is based on PPy NPs supported on liquid-phase exfoliated (LPE) graphene and constitutes an inexpensive, simple, and fast procedure to obtain graphene nanocomposites with suitable decoration.

An additional graphene-based solution was prepared to act as a reference sample. In this case, a bare graphene solution was prepared and deposited as mentioned above. In consequence, the effect of the sensing performance induced by the presence of PPy NPs can be elucidated by comparing the sensing results obtained using the reference sample (bare graphene) and the PPy-decorated one (PPy@Graphene).

As it was mentioned before, the PPy NP synthesis and graphene decoration were conducted using distilled water and room-temperature working conditions. Furthermore, the products obtained (PPy and graphene) are biocompatible, constituting a green experimental procedure.

### Sensing Device Fabrication and Gas-Sensing Measurements

The resulting graphene nanoflakes decorated with polypyrrole NPs were deposited via the airbrushing method, using nitrogen as a carrier gas while the hot plate (i.e., substrate holder) was kept at 115 °C. The solution was deposited onto alumina substrates that comprised platinum screen-printed electrodes [Figure S1, Supporting Information (SI)]. Subsequently, the gas sensors were placed in an airtight Teflon chamber with a volume of 35 cm^3^. The sensing chamber was connected to a gas mixing and delivery system that used calibrated gas cylinders and pure dry air (Air Premier purity: 99.999%) as carrier. Then, the resistance of the different sensors was monitored using a multimeter (HP 34972A, Agilent), and resistance changes were recorded while different concentrations of gases were applied. In order to reduce the power consumption of the system and to work under more realistic experimental conditions, the total flow was adjusted at a low rate (100 mL/min) using a set of mass-flow controllers (Bronkhorst High-Tech B.V.) and electrovalves. The sensors were stabilized under dry air for 15 min before being exposed to a given concentration of a gaseous species for 5 min. The responses to several concentrations were recorded by applying successive dilutions of gases and defining sensor response as Δ*R*/*R*_0_ expressed in percentage, where Δ*R* corresponds to the resistance changes recorded over 5 min of gas exposure, while *R*_0_ is given by the resistance of the sensor in air (or baseline).

In order to characterize the gas-sensing performance under a humid atmosphere, a controller evaporator mixer (Bronkhorst High-Tech B.V.) was used to humidify the atmosphere during some of the measurements. With that, the effect of the ambient moisture on sensor response was evaluated as well.

### Characterization of the Gas-Sensitive Materials

**Figure 1:**
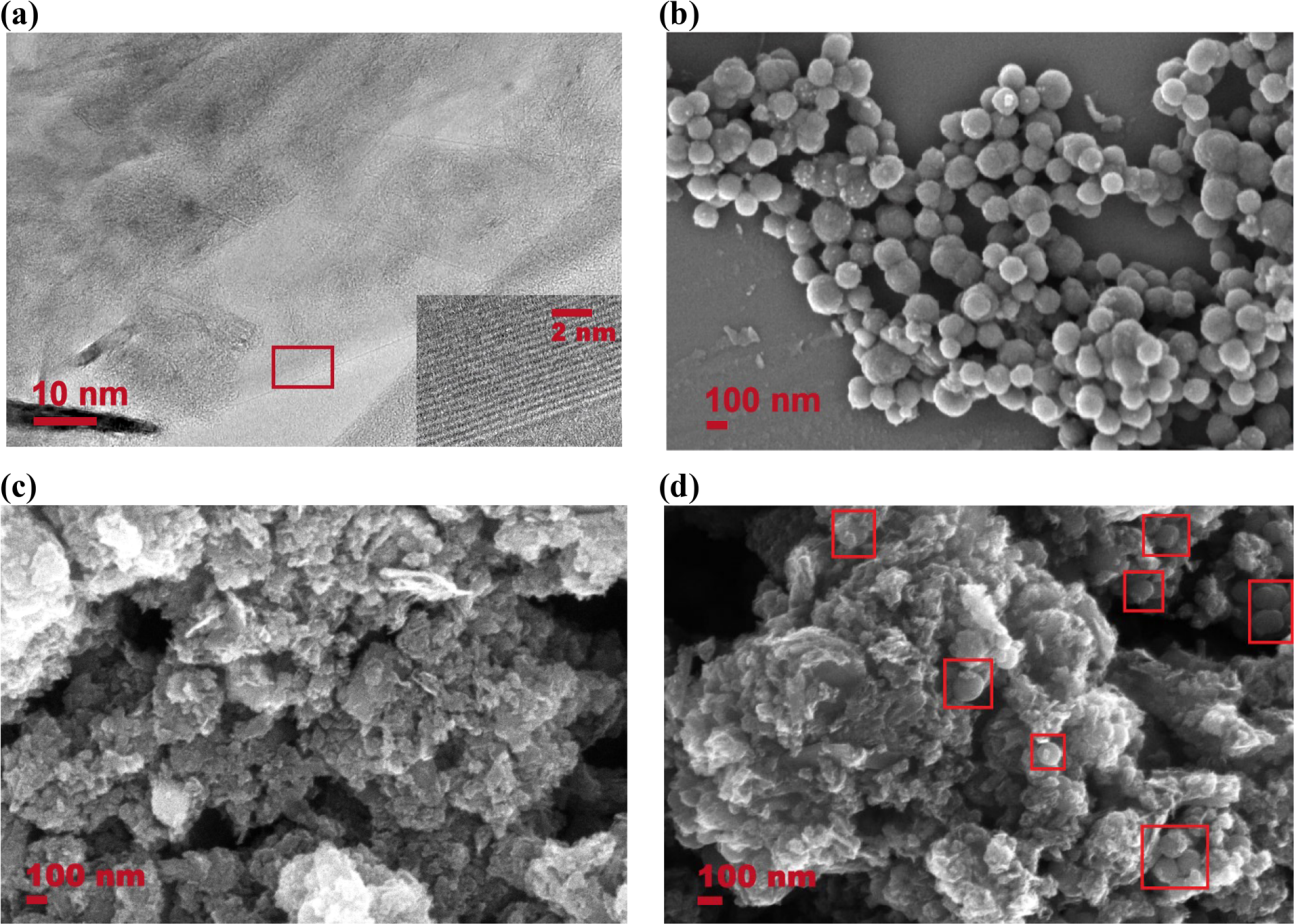
(a) HRTEM image of bare graphene; the inset shows a layered graphene structure (the red frame in the image indicates the area of the inset). FESEM images of synthesized polypyrrole nanoparticles (PPy NPs) (b), the surface of the bare graphene (c), and graphene nanoflakes decorated with PPy NPs (d) (red squares help to spot PPy nanoparticles on loaded graphene).

Physical and chemical properties of the as developed sensitive films were analyzed using several experimental techniques. The morphological features of PPy@Graphene were studied using a high-resolution transmission electron microscope (HRTEM) (JEM 2100, JEOL Ltd.). For TEM investigation of bare graphene, the layout deposited was scratched from an Al_2_O_3_ substrate and ultrasonically dispersed for 20 min, at 37 kHz and 100% of power, in a mixture of methanol and water. One drop of the dispersion was then deposited on a lacy carbon film supported by a copper grid (300 mesh). Figure 1a shows a section of a graphene flake, which is part of a larger structure (Figure S2a, SI) with a size of about 270 × 400 nm^2^. An image obtained at higher magnification (inset in Figure 1a) shows that this structure consists of about 20 graphene layers with an average interlayer distance of 0.4 nm.

An AG-Ultra 55 (Zeiss) field emission scanning electron microscope (FESEM) was used to analyze both nanomaterials separately and the resulting sensitive film. The synthesized PPy NPs suspended in water were deposited on a piece of a silicon wafer, showing monodispersed nanoparticles (Figure 1b). Besides, the histogram depicted in Figure S2b (SI) shows a narrow nanoparticle size distribution, situating the highest frequency in the range from 100 to 120 nm. Figure 1c shows the surface of the bare graphene layer. A highly porous surface can be appreciated, which usually leads to better sensing performances. Furthermore, Figure 1d depicts the graphene nanoflakes loaded with PPy NPs, demonstrating an appropriate decoration and nanoparticle distribution.

**Figure 2:**
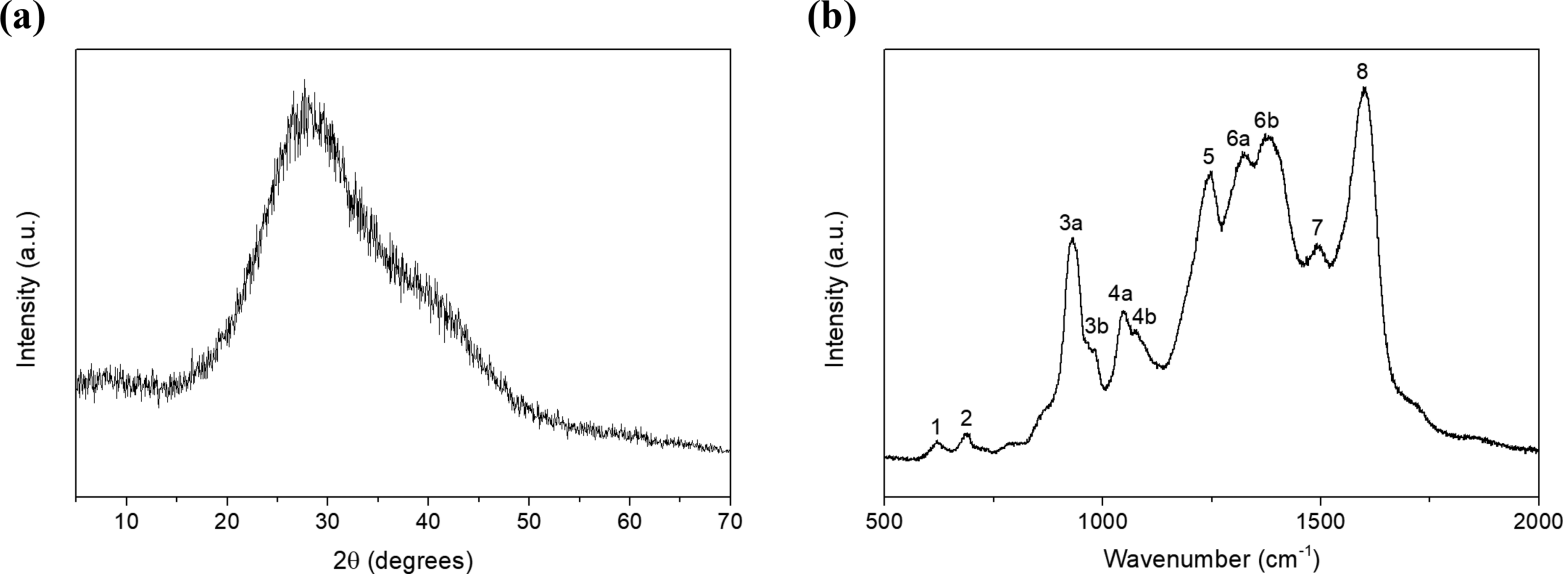
XRD pattern (a) and Raman spectrum (b) of polypyrrole nanoparticles. The Raman bands’ assignment is summarized in Table 1.

An X-ray diffraction (XRD) analysis was conducted using a Bruker-AXS D8-Discover diffractometer (Bruker Co.) with a parallel incident beam (Incoated GmbH). The X-ray diffractometer was equipped with a HI-STAR general area diffraction system (GAADS) detector from Bruker Co. The XRD measurement was conducted at 40 kV and 40 mA to generate the Cu Kα radiation, and the data were collected stepwise over the range 2θ = 5°–70°. Figure 2a shows the XRD pattern of PPy NPs, in which a characteristic broad peak appears at 2θ = 28.6°, revealing low crystallinity and the amorphous structure of PPy.^36^ This broadening is associated with the scattering of X-rays from the PPy chains at the interplanar spacing.^37^

The synthesized PPy NPs were also characterized by employing Raman spectroscopy. The PPy powder was placed onto a piece of a silicon wafer and the analysis was conducted using a Raman spectrometer (Renishaw plc) with a coupled confocal Leica DM2500 microscope (Leica Microsystems GmbH). The laser employed had a wavelength of 514 nm and the acquisition time was set to 30 s, while three cycles or repetitions were acquired over the spectra to reduce the noise level. Figure 2b shows characteristic bands of PPy NPs summarized in Table 1.

**Table 1:** Raman Bands for Polypyrrole Nanoparticles in Figure 2b

peak	wavenumber (cm^–1^)	band assignment
1	620	C–C ring (torsional)
2	688	C–H wagging
3a, 3b	930, 976	C–C ring deformation (bipolaron and polaron, respectively)
4a, 4b	1048, 1074	C–H in-plane deformation (polaron and bipolaron, respectively)
5	1245	antisymmetric C–H in-plane bending
6a, 6b	1324, 1377	C–C in-ring, antisymmetric C–N stretching (6b is also associated with C–H and N–H bending stretching)
7	1495	C–C, C=N stretching
8	1600	C=C in-ring, C–C inter-ring stretching

Oxidation levels of PPy have a significant influence on the electrical and physicochemical properties and, therefore, over the sensing performance. The PPy structure consists of a conjugated system, alternating single and double covalent bonds through the overlapping of the p orbitals with delocalized electrons (σ- and π-bonds). These oxidized states are associated with intermediate energy levels within the band gap, resulting in polarons and bipolarons.^38^ A polaron is formed when a π-electron is moved out from the neutral PPy, while a bipolaron can be formed when a second electron is removed. Both forms act as charge carriers along the conductive polymer.^39^ Thus, these electron relocations as polarons or bipolarons induce crystal lattice distortions in the PPy rings.^40^ Raman spectroscopy for bare PPy NPs (Figure 2b) reveals the characteristic bands related to several molecular interactions. The Raman fingerprint obtained is summarized in Table 1 and is consistent with those previously reported in the literature.^41,42^

**Figure 3:**
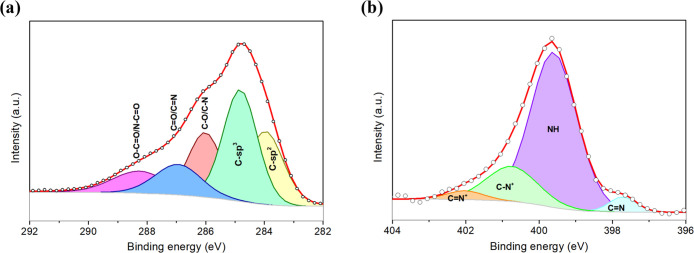
C 1s (a) and N 1s (b) XPS core level peaks obtained for the bare PPy NPs.

X-ray spectroscopy (XPS) has been conducted to analyze the chemical composition of synthesized PPy NPs using a VERSAPROBE PHI 5000 (Physical Electronics, Inc.) equipped with a monochromatic Al Kα X-ray source. High-resolution XPS spectra were recorded, accounting for an overall energy resolution of about 0.6 eV. Figure 3a shows the C 1s core level deconvoluted in several components, revealing as the most predominant one the carbon with sp^3^ configuration (C_α_) at 285.3 eV. This type of carbon bonding is associated with sp^3^ lattice defects and amorphous carbon, being consistent with the low crystallinity registered for the XRD analysis. The component centered at 284.4 eV reflects photoelectrons emitted from the sp^2^ carbon configuration (C_β_), which is related to aromatic carbon bonds, whereas the components shifted to higher binding energies are attributed to the C–N (or C–O), C=N (or C=O), and N–C=O (or O–C=O), appearing at 286.5, 287.4, and 288.7 eV, respectively. The components related to oxygen-containing functional groups (also observed in the O 1s core level, Figure S3, SI), such as carbonyl and carboxyl terminations, probably originated during PPy polymerization, in which the terminations of the chains can interact with water to form the oxygen groups.^43^

The deconvolution of the N 1s core level from PPy NPs is depicted in Figure 3b. This peak shows four components, with an intense peak at 400.0 eV associated with neutral nitrogen (N–H structure) in the PPy ring, whereas the component at the lowest energy binding (398.1 eV) is related to imine structures (C=N). Besides, at higher binding energies, two components appear related to the positively charged nitrogen, which are polarons (C–N^+^) and bipolarons (C=N^+^) at 401.2 and 402.5 eV, respectively. As a result, polarons are more predominant than bipolarons in the synthesized PPy NPs.

Nitrogen adsorption–desorption analysis of bare and PPy-decorated graphene was conducted using a Quadrasorb SI surface analyzer (Quantachrome Instruments) for determining the BET (Brunauer–Emmett–Teller) area, average pore size, and pore volume. The samples were degassed overnight at 120 °C before this measurement. The specific surface area was calculated through the BET method and the pore size distribution was determined by the BJH (Barret, Joyner, and Halenda) method. The obtained N_2_ adsorption/desorption results are summarized in Figure S4 and Table S1 (SI). Specifically, the BET area obtained for bare graphene (730 m^2^/g) was slightly higher than PPy@Graphene (644 m^2^/g). This result can be expected since PPy NPs probably present a significantly lower BET area. Nevertheless, negligible changes in the average pore diameters and pore volumes were observed between both samples, revealing that these parameters are mainly ruled by graphene nanoplatelets.

## Results and Discussion

**Figure 4:**
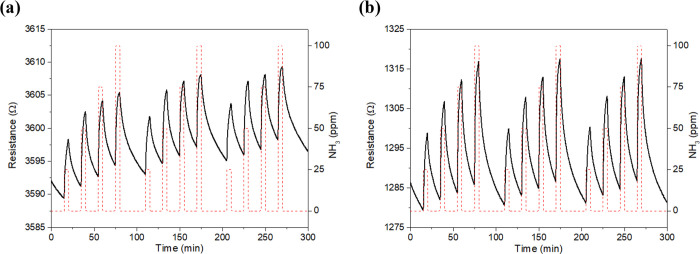
Example of NH_3_ detection (range 25–100 ppm) at room temperature for bare (a) and PPy NPs-loaded (b) graphene samples (resistance, black line and left *y*-axis; gas concentration, red dashed line and right *y*-axis).

The ability of the hybrid nanomaterial developed for detecting NH_3_ was evaluated in similar conditions to those needed in real-time monitoring. First measurements consisted of repeated response and recovery cycles to 25, 50, 75, and 100 ppm of NH_3_. Figure 4 shows the typical dynamic responses obtained for the bare (Figure 4a) and the PPy-loaded graphene films, which experienced a significant increase in their resistance when exposed to the target gas. Figure 4b, which corresponds to the polymer-decorated graphene, shows that this hybrid nanomaterial shows higher repeatability and better baseline stability than its bare counterpart (Figure 4a). Calibration curves of both types of sensors are summarized in Figure S5 (SI), showing that graphene decorated with PPy offers up to 7-fold higher responses (i.e., intensity of the resistance changes induced by the exposure to ammonia) than bare graphene. Besides, since sensitivity is given by the slope of the calibration curves, PPy@Graphene presents 14 times higher sensitivity than bare graphene.

Conjugated polymers are well-known semiconductor materials, whereas PPy stands out due to the high positions of its HOMO and LUMO levels, being easily p-doped and conferring high stability.^44^ In consequence, both nanomaterials employed, oxygen-defective graphene and Cl^–^-doped PPy, show a mild p-type behavior. When the sensitive layer interacts with an electron-donating molecule such as NH_3_, the hole density is lowered, thus resulting in an increased electrical resistance. According to the experimental findings, it can be assumed that bare graphene can interact with NH_3_ molecules; however, the presence of PPy NPs decorating this carbon nanomaterial involves a more intense interaction. In other words, the presence of both nanomaterials creates a synergistic sensing effect, in which graphene and PPy are interacting with the target gas, thus resulting in superior sensing performance. Besides, both nanomaterials show a noteworthy interaction through strong hydrogen bonds and π–π-stacking.^45^ As a result, long-term sensor stability can be attributed to their efficient interaction.^46^ In addition, the large graphene surface area and its superior electron mobility induce a fast and efficient charge transfer with PPy NPs, leading to an effective transduction.^47^

The somewhat limited interaction between graphene and NH_3_ is well-known, especially under room-temperature conditions.^48^ In particular, oxygenated defects and functional groups grafted at graphene act as adsorption sites for ammonia molecules, leading a charge transfer toward the sensitive film as follows:
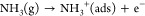
1

Nevertheless, PPy NPs tend to show higher interaction with NH_3_, thus resulting in better sensing performance. Overall, when graphene nanoflakes are decorated with PPy, these NPs contribute to the NH_3_ sensing through the donation of a lone pair of electrons of nitrogen from the target gas to the initially oxidized PPy, which results in a neutralized PPy through the reduction of their doping level, thus decreasing conductivity. Indeed, the ammonia–polymer interaction is based on a compensation effect, in which the doping level of the polymer chains varies through two reversible pathways.^49^ The first one is related to an electron transfer, in which the positively charged PPy NPs (PPy^+^) are reduced to their neutral form (PPy^0^) when interacting with NH_3_ molecules, whereas the dopant anion (Cl^–^) that was compensating the charged PPy^+^ is reversibly displaced as follows:^50^
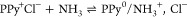
2

The second feasible sensing mechanism involves a proton transfer between ammonia, which is a strong base, with the PPy NPs. Specifically, the PPy deprotonation toward ammonia results in the formation of the ammonium ion that induces a compensation effect through the dopant anion. Therefore, the doping level is reduced as follows:^50^
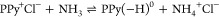
3

**Figure 5:**
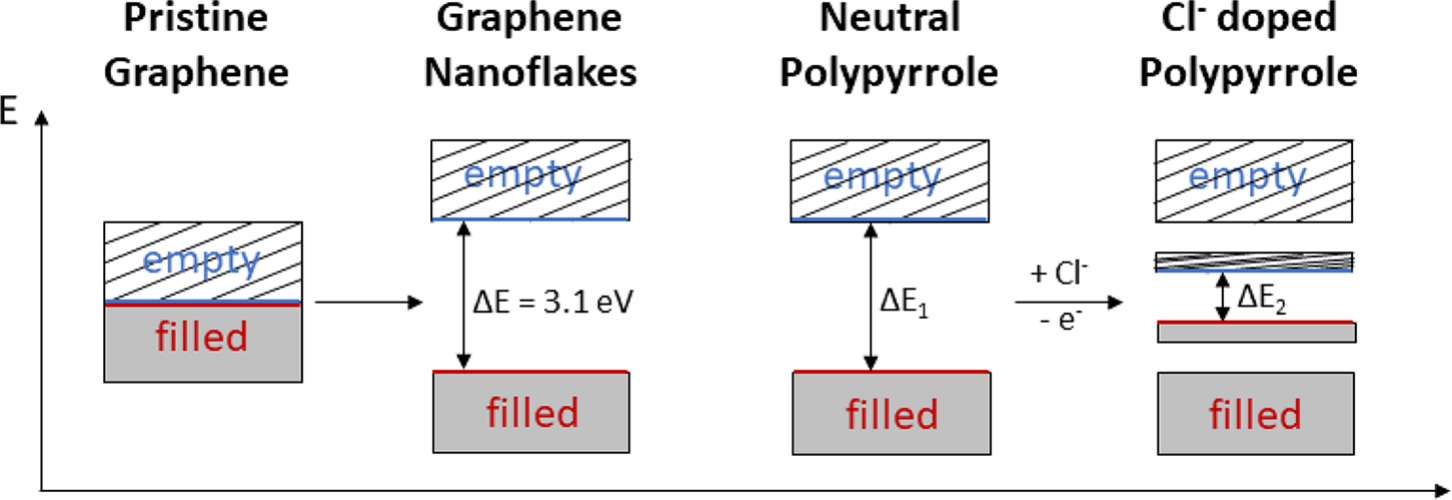
Band gap variation between ideal pristine graphene and the nanoflakes employed in this work and the band gap reduction of doped PPy NPs in comparison to their neutral counterparts.

It is worth noting that, roughly, greater specific BET areas usually led to improved sensing performance owing to a larger area for adsorbing gas species.^51^ However, graphene decorated with PPy NPs showed better sensing performance than bare graphene despite its lower BET area. This fact is revealing the essential role of PPy in the NH_3_ gas detection, enabling a significant electronic sensitization that compensates this lower specific area and confirming the importance of the sensing mechanisms proposed. Nevertheless, the mere presence of the nanoparticles cannot explain the outstanding enhancement in the sensing responses to NH_3_. Therefore, a synergistic effect between graphene and PPy is probably held. For that reason, their interface has been extensively studied. Roughly, both nanomaterials are composed of sp^2^ conjugated carbons resulting in pristine graphene and neutral polypyrrole, showing semimetallic behavior. Theoretically, pristine graphene is a zero band gap semiconductor with a tiny overlap between the valence and conduction bands.^52^ Nonetheless, XPS analysis revealed that graphene employed in this work shows a significant oxygen content (owing to different oxygen functional groups present at the surface), which results in p-type doping. This chemical functionalization and the presence of the sp^3^ carbon configuration induce a band gap opening in graphene (Figure 5). Indeed, a UV–visible absorption spectrum was recorded for graphene nanoflakes (Figure S6a, SI) using a Cary 5000 UV–vis–NIR spectrophotometer (Agilent). Subsequently, the band gap was determined through a Tauc plot, in which the gap energy was calculated by extrapolating the linear portion of the graph between the function (α*h*ν)^2^ versus the photon energy (eV), as shown in Figure S6b (SI). A relatively wide band gap (3.1 eV) for graphene nanoflakes resulted.

Regarding the PPy, this nanomaterial is a well-known conducting polymer, but a large band gap separates the highest occupied molecular orbital (HOMO) and the lowest unoccupied molecular orbital (LUMO). Specifically, previous works report a 3.2 eV band gap for PPy in its neutral state,^53^ resulting in poor electronic conductivity. Nevertheless, it is possible to increase the electronic transport by adding new energy levels within this band gap (Figure 5). Indeed, polymer doping induces significant changes in the electronic profile due to the electron removal or addition from the chain. The synthesis route and reagents have a key influence in this doping. Thus, in the present study PPy NPs doped with Cl^–^ were obtained due to the use of FeCl_3_ as an oxidizing agent. At low doping density, the polaron energy states raise the HOMO and LUMO levels away from both sides of the band gap. In other words, the HOMO level shifts up while the LUMO level shifts down. But as Figure 2b depicts, bipolarons are also present in the synthesized PPy NPs. As a result, polaron states are partially combined, leading to bipolaron energy states, which are shifting the energy levels far away from either edge of the gap. In consequence, when these energy bands overlap, new intermediate band structures are formed at the polymer, significantly reducing the band gap down to 1.5 eV,^54^ resulting in a more efficient and straightforward electronic transport throughout the PPy NPs.^55^

**Figure 6:**
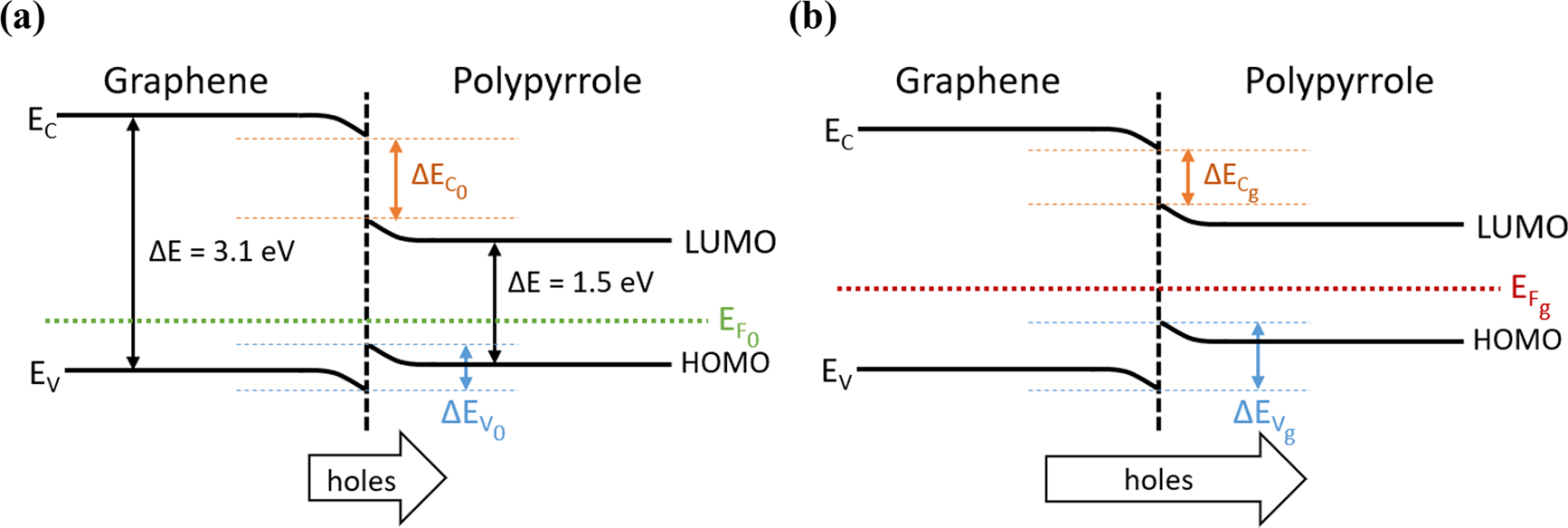
Band diagram showing the p–p isotype junction between graphene and PPy NPs in air (a) and when is exposed to NH_3_ gas (b). The Fermi level shifts up under NH_3_ exposure (*E*_F_g__) in comparison to in air (*E*_F_0__).

Figure 6a depicts a summary of the energy level diagrams for graphene and PPy NPs. This p–p isotype junction, at equilibrium under ideal conditions, shows greater discontinuity in the conduction bands (Δ*E*_C_) relative to the valence bands (Δ*E*_V_), owing to the closer levels of valence bands between the two nanomaterials according to XPS analysis.^56^ Therefore, excitations of the sensitive layer translate into positive charges (holes) being emitted from the graphene over the barrier into the PPy NPs. In consequence, near the boundary of the interface a depletion region can be formed,^57^ specifically located on the graphene surrounding PPy. Conversely, PPy NPs probably increase carrier concentration, thus being considered as accumulation regions. As a result, the PPy@Graphene sensing performance was enhanced because the NPs would have an excess of positive charges that can be, to some extent, recombined with the negative charges when interacting with electron donor gas molecules such as NH_3_. Meanwhile, graphene is lowering its concentration of majority charge carriers (holes), further decreasing its conductivity and enhancing gas-sensing response. This fact is worth mentioning considering that graphene is mainly acting as a transducer (since limited interaction with gases cannot be ruled out) while the main gas interaction is probably located at the PPy NPs, acting as gas recognition elements. Indeed, the potential applied to the transducer element (graphene) is increasing the charge flow and in consequence favoring the hole movement from graphene to PPy NPs over the barrier.

Considering that NH_3_ is a donor of electronic charge while the sensitive film is the receptor, the HOMO level of the gas molecule interacts with the LUMO level of the sensor.^58^Figure 6b depicts a schematic energy band diagram of the graphene/polypyrrole interface when exposed to a strongly nucleophilic molecule like NH_3_. This electron-donating gas specie is attached to the sensitive film via coupling π-bonds.^59^ As a result, once the gas donates electrons to the PPy@Graphene layer, the Fermi level in dry air conditions (*E*_F_0__) undergoes an upward shift, reaching *E*_F_g__, which results in the lowering of the majority carrier (holes) concentration.

This shift in the Fermi level for graphene and PPy NPs is associated with a band-bending readjustment when the sensor is exposed to NH_3_. In consequence, once ammonia adsorbs at the hybrid nanomaterial, the energy gap at the interface between the valence band of graphene and the HOMO of PPy NPs widens (i.e., Δ*E*_V_g__ > Δ*E*_V_0__), which favors holes from the valence band of graphene to transfer toward the HOMO of PPy NPs. In other words, upon exposure to ammonia, the graphene layer loaded with PPy NPs sees the concentration of its majority charge carriers (holes) reduced, which translates into a significant change in the overall sensor resistance.

**Figure 7:**
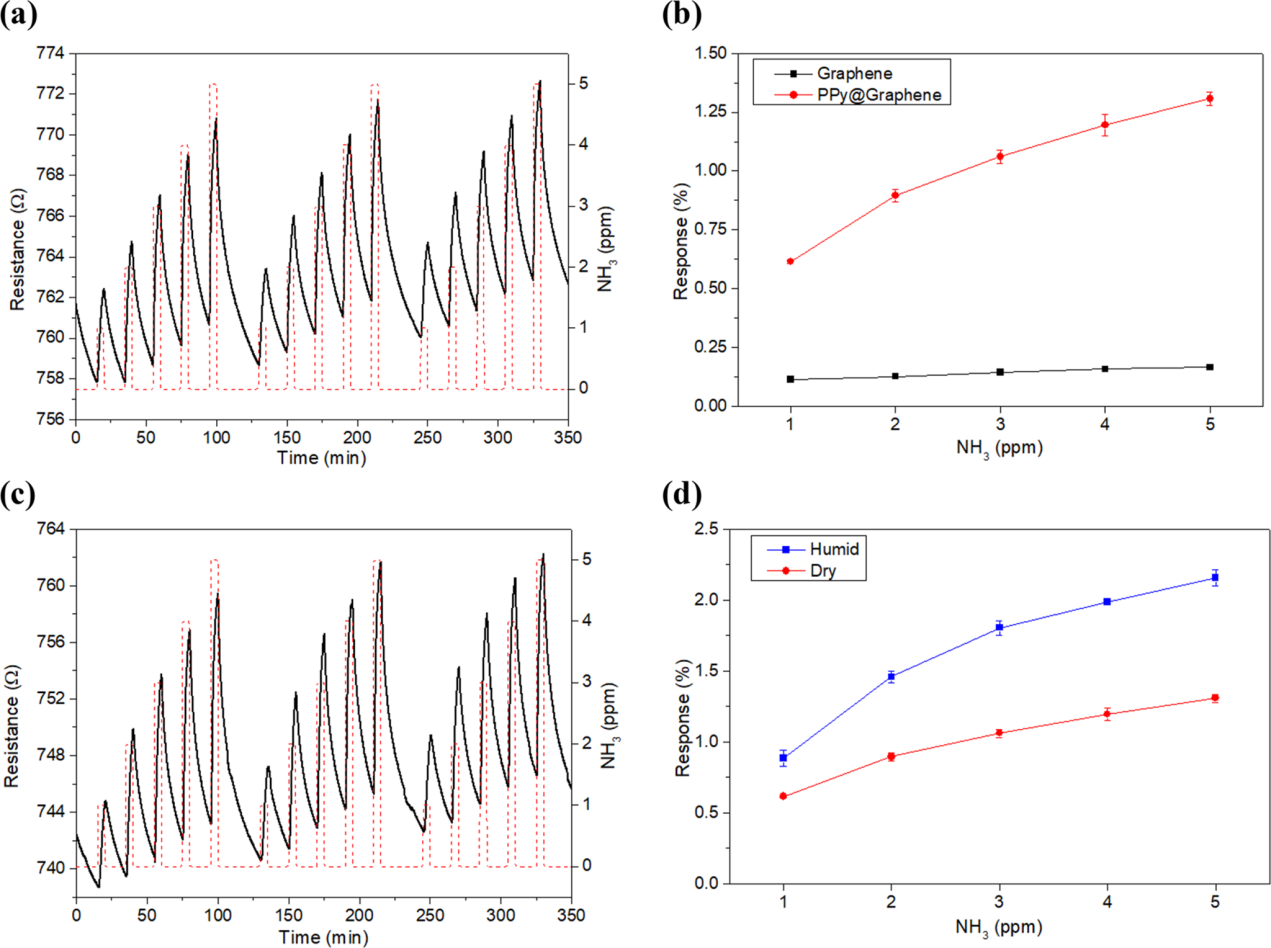
Examples of electrical responses when detecting NH_3_ in dry (a) and humid (c) conditions in the range of 1–5 ppm. Calibration curves obtained for bare and PPy NPs-loaded graphene at low NH_3_ concentrations (b). Comparison of the calibration curves obtained for a PPy@Graphene sample in a dry and humid atmosphere (d). For the experiments carried out in a humid atmosphere, the relative humidity was set up to 50%.

Considering the very low noise levels achieved in the detection of NH_3_ in the range of 25–100 ppm and the improved sensing performance when graphene is loaded with PPy NPs, further measurements at decreased target gas concentrations were performed. The intermediate range of ammonia concentrations ranging from 5 to 25 ppm was studied, and results can be found in the Supporting Information. Figure S7 (SI) shows the sensing responses and calibration curves obtained. It is worth noting that this range of concentration has significant interest for monitoring ammonia in the ambient because it is close to the TWA and STEL values defined by the agency NIOSH. Figure 7a shows the dynamic response of a PPy@Graphene sample for the detection of very low NH_3_ concentration levels (from 1 to 5 ppm). Even at this range of concentrations, a significant response repeatability and baseline stability was achieved under room-temperature operation conditions. Conversely, under these experimental conditions, the bare graphene sample shows a significant baseline drift (Figure S8a, SI) and poor sensitivity. Indeed, Figure 7b depicts a clear enhancement of the sensing performance when graphene is decorated with PPy NPs. Specifically, PPy@Graphene shows higher resistance changes (6-fold) and sensitivity (13-fold) than bare graphene.

Nevertheless, the presence of ambient moisture is a key influencing factor on the gas-sensing performance due to its interfering effect. In consequence, the previous experiments carried out under dry conditions were reproduced under humid conditions (50% relative humidity at 23 °C). Figure 7c shows the dynamic response of the decorated graphene, being quite similar to those obtained under dry conditions (Figure 6a). However, as Figure 7d depicts, PPy@Graphene samples show slightly higher responses under humid conditions than in a dry atmosphere. Specifically, the response under humid conditions was 60% higher, while the sensitivity was increased 1.6-fold. As expected, PPy@Graphene samples increase their response toward ammonia in the presence of moisture. When water molecules get adsorbed on the PPy a swelling effect in the polymer chains can occur. As a result, the polymer network shows a greater distance between their chains, resulting in a further increase in resistance.^60^ In addition, adsorbed water molecules might enhance the proton exchange with the NH_3_ molecule, inducing a further decrease in the hole concentration, thus leading to higher resistance levels.

However, since these higher sensing responses can be beneficial to lower the LOD or improve other sensing parameters, variable relative humidity levels registered under real conditions of operation can constitute a significant drawback. To solve that, an alternative might be the creation of a sensor array, in which a specific humidity sensor is integrated. Therefore, with the calibration of the PPy@Graphene sensor at multiple ambient moisture levels, this issue could be overcome.

**Figure 8:**
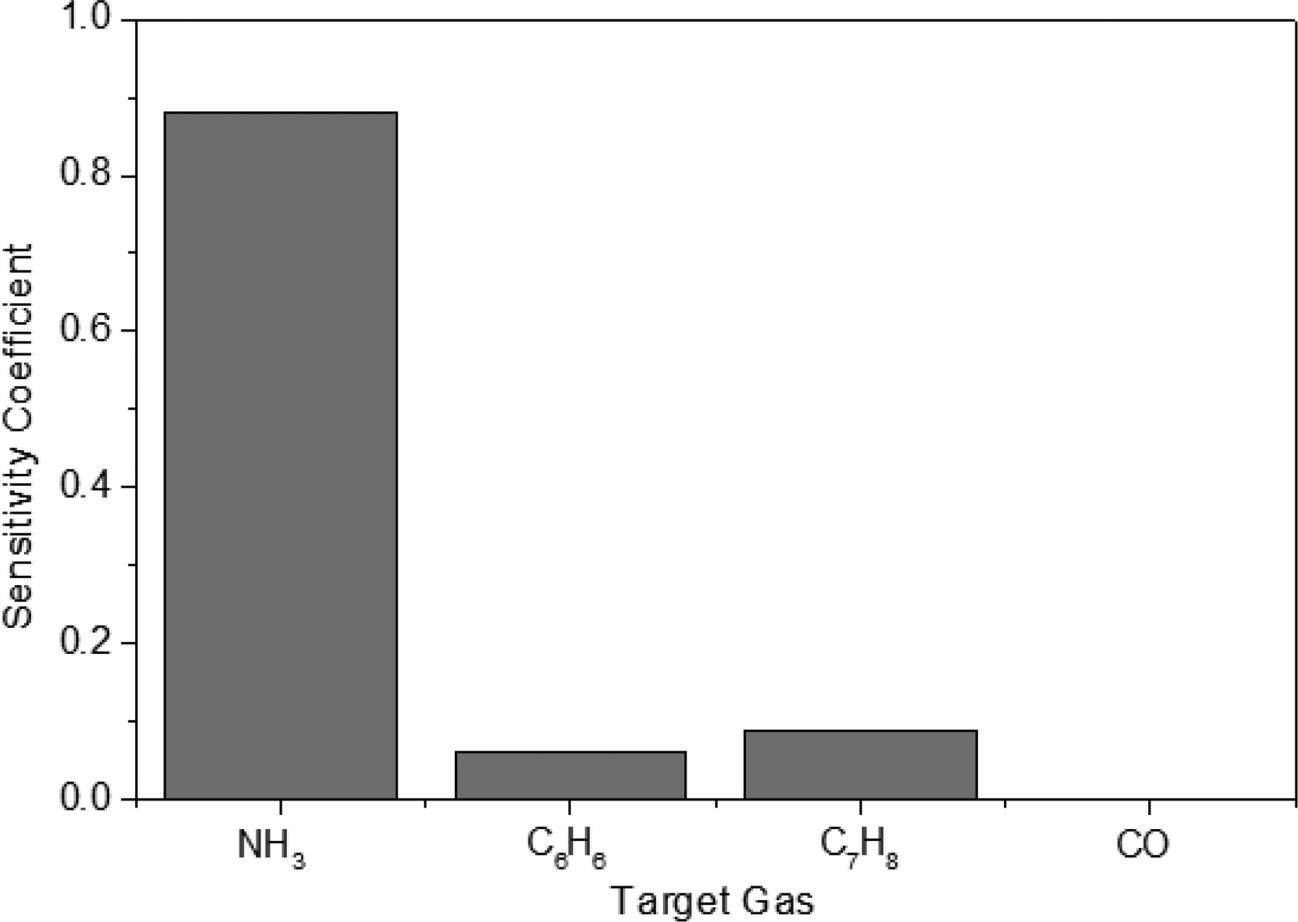
Sensitivity coefficients (expressed as response/concentration) for the different gas compounds tested.

Considering the gas-sensing results obtained under a humid atmosphere, the limit of detection (LOD) was estimated through the following equation
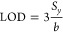
4where S_*y*_ is the standard deviation of *y*-residuals and *b* corresponds to the sensitivity (slope) from the calibration curve. As a result, the PPy@Graphene shows a LOD of 491 ppb for NH_3_, which is comparable to other types of organic-based nanomaterials. For instance, the use of a conductive sublayer of perfluoro-copper phthalocyanine (Cu(F16Pc) covered by a lutetium bisphthalocyanine (LuPc2) sensitive layer reveals a LOD of 140 and 2000 ppb depending on if DMBz or TFBz was respectively grafted to the ITO substrate.^61^ Conversely, Figure S8b (SI) shows a significant baseline drift and poor sensing responses for bare graphene samples, denoting the enhancement effect of PPy NPs decorating graphene nanoflakes. Furthermore, cross-sensitivity was assessed (Figure 8) by measuring other reducing species like carbon monoxide (CO) and aromatic volatile organic compounds (VOCs) such as benzene (C_6_H_6_) and toluene (C_7_H_8_). As a result, negligible resistance changes were obtained toward these analytes in comparison to NH_3_, making the PPy@Graphene gas sensor reliable and promising to detect ammonia in the environment.

**Figure 9:**
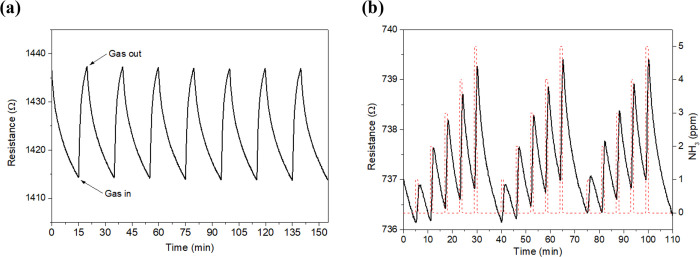
Repeatability test for a PPy@Graphene sensor by applying successive exposures of 50 ppm of NH_3_ (a). Fast NH_3_ detection (gas exposure of 1 min) by employing a PPy@Graphene sensor (b).

To better understand the potential of the as developed hybrid nanocomposite material, additional tests were performed to assess the sensing performance of graphene nanoflakes decorated with polypyrrole nanoparticles. The sensor repeatability was evaluated by applying successive NH_3_ pulses for 5 min and recovery steps of 15 min between gas exposures. Figure 9a shows an excellent sensor repeatability, in which the hybrid nanomaterial PPy@Graphene presents an error of about 0.7%. Besides, since fast detection might be needed in ambient monitoring applications, the sensor responses to NH_3_ were also evaluated by reducing the exposure time from 5 to 1 min, while the duration of recovery steps was lowered from 15 to 5 min (Figure 9b). Even under these experimental conditions, clear and repeatable sensor responses were achieved.

Despite these superior sensing properties, a demonstration of high stability and durability is also required before the potential implementation of this sensor in commercial devices. For that reason, a stability test was performed by measuring and comparing the sensing responses toward 50 ppm of ammonia for 5 months (Figure S9, SI). Only a small decrease in response was registered after this 5-month period. Nevertheless, to better our understanding, the sensing results were correlated with an XPS analysis performed on samples freshly synthesized and after 5 months of continuous use, in order to assess aging.

**Figure 10:**
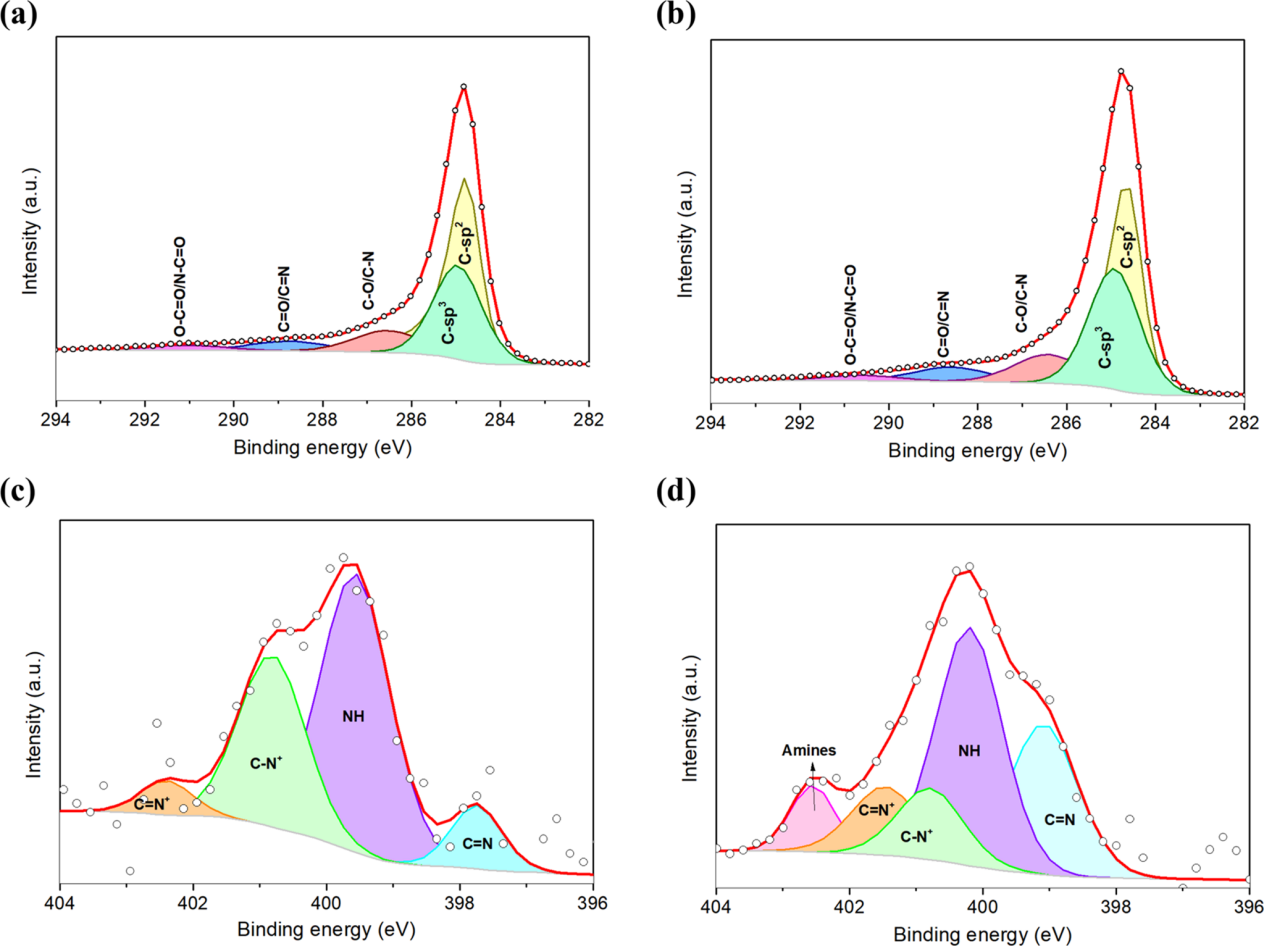
C 1s core levels obtained for the sample PPy@Graphene before (a) and after (b) NH_3_ gas sensing. Similarly, the N 1s were assessed before (c) and after (d) the successive ammonia exposures.

The C 1s core levels were analyzed before the NH_3_ gas sensing (Figure 10a) and after 5 months of use (Figure 10b). Table S2 (SI) summarizes the relative areas of the different components, showing the absence of significant differences between both C 1s spectra. Indeed, just a slight decrease in the sp^2^ configuration can be observed, while a limited increase in both the sp^3^ carbon configuration and oxygen content through a higher intensity peak of C–O/C–N and C=O/C=N is obtained. These results indicate a slight aging of the sensitive film after 5 months due to the slight oxidation of the hybrid nanomaterial, demonstrating superior reusability and stability. N 1s core levels studied on the PPy@Graphene sample before (Figure 10c) and after (Figure 10d) NH_3_ sensing are consistent with those assessed for the C 1s core levels. Particularly, the quantification of the relative concentration of the components shows a decrease in the neutral nitrogen (NH) and an increase in the C=N defects of the PPy due to the loss of conjugation during aging (Table S3, SI). Interestingly, at higher binding energy a new component appears when the sensor is exposed to NH_3_ (Figure 10d). In fact, this peak can be associated with the presence of amines, denoting NH_3_ adsorbed on the sensitive layer. In addition, the O 1s core level component was also analyzed before and after NH_3_ sensing (Figure S10) for a PPy@Graphene sample. The analysis of the O 1s spectrum shows an oxygen content related to the partial oxidation of PPy and the oxygen-containing groups grafted to graphene. Interestingly, a decrease in the abundance of the C=O bond was observed while C–O experiences a significant increase. This fact can be derived from the conjugation of carbonyl groups due to the oxidation of PPy and the interaction with the gaseous environment.

Therefore, the low-rate decay of response intensity with time and its consequent low aging rate could be attributed to several reasons. The PPy NPs synthesis using FeCl_3_ as an oxidant element leads to higher stability of electrical conductivity than other oxidants such as Fe_2_(SO_4_)_3_ or (NH_4_)_2_S_2_O_8_.^62^ The reason is that some anions like SO_4_^–2^ tend to experience higher migration to pyrrole nitrogen than Cl^–^ anions, resulting in the quaternization of the nitrogen, thus increasing the PPy degradation and instability. Besides, since sunlight and operating conditions well above room temperature are well-known parameters that induce the aging and degradation of PPy,^63,64^ the gas-sensing measurements have been conducted under dark conditions and ambient temperature. Both parameters can be easily implemented in airtight testing chambers, favoring the preservation and stability of PPy NPs. Even more noticeably, graphene probably plays an essential role in preventing PPy aging thanks to its high hydrophobicity. When water molecules directly interact with PPy chains, the electron density of nitrogen atoms is changed,^65^ weakening the interaction between the PPy and the dopant anion, in this case, Cl^–^. As a result, ambient moisture reduces the electrical conductivity of PPy NPs, accelerating their aging. However, the hydrophobic character of graphene may protect the PPy NPs toward their aging derived from the interaction with water molecules. This fact was already demonstrated for other types of sensitive nanomaterials, such as halide perovskites, in which graphene increases their lifetime and stability toward the presence of relative humidity in gas sensors^66^ and solar cells.^67^

Finally, Table S4 (SI) is summarizing several works that report the use of different PPy/graphene hybrid configurations. Overall, an increase in the sensing performance was achieved when the PPy is present in the sensitive film. However, essential information is frequently missing, such as the flow rate applied, LOD, and different tests to assess the repeatability, stability, and effect of the ambient moisture. Table S4 (SI) depicts that graphene nanoflakes loaded with PPy NPs show an outstanding sensitivity coefficient within the works that report an in situ chemical polymerization. It is worth noting that electrochemical polymerization can lead to better sensing responses, since high control over the thickness and PPy geometry can be achieved. However, their use involves more expensive synthesis procedures and fewer possibilities for industrial-scale production. Besides, Table S4 (SI) shows that the present work uses a low flow rate, 10-fold lower than those reported in other works, as well as the use of air instead of nitrogen as carrier gas. With these experimental parameters, the sensing performance is probably underestimated to some extent because higher flow rates or sensing in a nitrogen atmosphere can boost up the resistance changes. But experimental conditions applied here are closer to those needed in real-time monitoring of ambient pollutants.

## Conclusion

A solvent-free, green method was proposed to obtain a graphene nanocomposite loaded with polypyrrole nanoparticles for detecting NH_3_ under ambient temperature operating conditions. The chemical polymerization of PPy NPs offers a low-cost and mass-scalable synthesis method, resulting in biocompatible nanomaterials with outstanding electrical properties. Indeed, the development of a chemical resistive device able to operate at room temperature paves the way for achieving widespread air quality monitoring systems due to their simple readout interface circuitry, inexpensiveness, low-power consumption, miniaturization, and high portability. Besides, high gas sensitivity and remarkable repeatability and LOD were obtained for detecting NH_3_. The sensing mechanisms are probably dominated by electron transfer, in which the presence of PPy decorating the graphene nanoflakes induces a larger and more effective charge transfer interaction, leading to enhanced sensing properties.

Nevertheless, despite the reduced footprint of this technology, high stability over time is mandatory for gas sensors. In consequence, the sensing properties were assessed for 5 months, and XPS analysis was conducted to study the aging of the hybrid nanomaterial. After an intensive use of the developed sensors, a suitable stability was observed. The slight oxidation of PPy observed in the XPS analysis is in agreement with the slight decrease registered in response intensity after several months of use in humid atmospheres. These results indicate that these sensors meet many of the specifications for being employed successfully in ambient monitoring applications.

Noteworthy, the present work reports the first use of a nanocomposite comprising PPy NPs morphology and graphene, revealing an outstanding sensing performance owing to the significant synergistic effect derived from the integration of both nanomaterials. With all of these, the combination of both graphene and PPy NPs is paving the way toward low power consumption and highly durable devices thanks to their capability to be operated at ambient temperature. Thereby, the as developed graphene loaded with PPy NPs shows unprecedented sensing properties, increasing its potential to be employed in commercial devices. However, further optimizing this hybrid gas-sensitive material (e.g., by fine-tuning the loading level of graphene with PPy NPs or adjusting the size of PPy NPs) will be the subject of future research.

## Supporting Information Available

Photograph of PPy@Graphene layer deposited on alumina substrate, HRTEM images, XPS spectra, UV–vis absorption spectrum, band gap plot, gas-sensing calibration curves, electrical responses detecting NH_3_, stability study, XPS quantification, XPS O 1s core level before and after sensing, and comparison of the sensor performances with those from the literature (PDF)

## References

[ref1] Reames T. G.; Bravo M. A. People, place and pollution: Investigating relationships between air quality perceptions, health concerns, exposure, and individual- and area-level characteristics. Environ. Int. 2019, 122, 244–255. 10.1016/j.envint.2018.11.013. 30449629

[ref2] West J. J.; Cohen A.; Dentener F.; Brunekreef B.; Zhu T.; Armstrong B.; Bell M. L.; Brauer M.; Carmichael G.; Costa D. L.; Dockery D. W.; Kleeman M.; Krzyzanowski M.; Künzli N.; Liousse C.; Lung S. C. C.; Martin R. V.; Pöschl U.; Pope C. A.; Roberts J. M.; Russell A. G.; Wiedinmyer C. What We Breathe Impacts Our Health: Improving Understanding of the Link between Air Pollution and Health. Environ. Sci. Technol. 2016, 50, 4895–4904. 10.1021/acs.est.5b03827. 27010639 PMC12303222

[ref3] González E.; Casanova-Chafer J.; Romero A.; Vilanova X.; Mitrovics J.; Llobet E. LoRa Sensor Network Development for Air Quality Monitoring or Detecting Gas Leakage Events. Sensors 2020, 20, 6225 10.3390/s20216225. PMC7672618 33142820

[ref4] Neri G. First Fifty Years of Chemoresistive Gas Sensors. Chemosensors 2015, 3, 1–20. 10.3390/chemosensors3010001.

[ref5] Regmi B. P.; Agah M. Micro Gas Chromatography: An Overview of Critical Components and Their Integration. Anal. Chem. 2018, 90, 13133–13150. 10.1021/acs.analchem.8b01461. 30359512

[ref6] Baron R.; Saffell J. Amperometric Gas Sensors as a Low Cost Emerging Technology Platform for Air Quality Monitoring Applications: A Review. ACS Sensors 2017, 2, 1553–1566. 10.1021/acssensors.7b00620. 29025261

[ref7] Jian Y.; Hu W.; Zhao Z.; Cheng P.; Haick H.; Yao M.; Wu W. Gas Sensors Based on Chemi-Resistive Hybrid Functional Nanomaterials. Nano-Micro Lett. 2020, 12, 71 10.1007/s40820-020-0407-5. PMC7770957 34138318

[ref8] Degler D.; Weimar U.; Barsan N. Current Understanding of the Fundamental Mechanisms of Doped and Loaded Semiconducting Metal-Oxide-Based Gas Sensing Materials. ACS Sensors 2019, 4, 2228–2249. 10.1021/acssensors.9b00975. 31365820

[ref9] Wang C.; Yin L.; Zhang L.; Xiang D.; Gao R. Metal Oxide Gas Sensors: Sensitivity and Influencing Factors. Sensors 2010, 10, 2088–2106. 10.3390/s100302088. 22294916 PMC3264469

[ref10] Sun D.; Luo Y.; Debliquy M.; Zhang C. Graphene-enhanced metal oxide gas sensors at room temperature: A review. Beilstein J. Nanotechnol. 2018, 9, 2832–2844. 10.3762/bjnano.9.264. 30498655 PMC6244217

[ref11] Alzate-Carvajal N.; Luican-Mayer A. Functionalized Graphene Surfaces for Selective Gas Sensing. ACS Omega 2020, 5, 21320–21329. 10.1021/acsomega.0c02861. 32905337 PMC7469114

[ref12] Ghanbari R.; Safaiee R.; Sheikhi M. H.; Golshan M. M.; Horastani Z. K. Graphene Decorated with Silver Nanoparticles as a Low-Temperature Methane Gas Sensor. ACS Appl. Mater. Interfaces 2019, 11, 21795–21806. 10.1021/acsami.9b00625. 31120237

[ref13] Casanova-Chafer J.; Garcia-Aboal R.; Atienzar P.; Llobet E. The role of anions and cations in the gas sensing mechanisms of graphene decorated with lead halide perovskite nanocrystals. Chem. Commun. 2020, 56, 8956–8959. 10.1039/D0CC02984J. 32638744

[ref14] Yuan W.; Shi G. Graphene-based gas sensors. J. Mater. Chem. A 2013, 1, 10078–10091. 10.1039/c3ta11774j.

[ref15] Mhlongo G. H.; Motaung D. E.; Cummings F. R.; Swart H. C.; Ray S. S. A highly responsive NH_3_ sensor based on Pd-loaded ZnO nanoparticles prepared via a chemical precipitation approach. Sci. Rep. 2019, 9, 9881 10.1038/s41598-019-46247-z. 31285474 PMC6614408

[ref16] Kim S.; Kwak D. H.; Choi I.; Hwang J.; Kwon B.; Lee E.; Ye J.; Lim H.; Cho K.; Chung H.-J.; Lee W. H. Enhanced Gas Sensing Properties of Graphene Transistor by Reduced Doping with Hydrophobic Polymer Brush as a Surface Modification Layer. ACS Appl. Mater. Interfaces 2020, 12, 55493–55500. 10.1021/acsami.0c17225. 33233877

[ref17] Li Q.; Chen D.; Miao J.; Lin S.; Yu Z.; Han Y.; Yang Z.; Zhi X.; Cui D.; An Z. Ag-Modified 3D Reduced Graphene Oxide Aerogel-Based Sensor with an Embedded Microheater for a Fast Response and High-Sensitive Detection of NO_2_. ACS Appl. Mater. Interfaces 2020, 12, 25243–25252. 10.1021/acsami.9b22098. 32391684

[ref18] Behi S.; Bohli N.; Casanova-Cháfer J.; Llobet E.; Abdelghani A. Metal Oxide Nanoparticle-Decorated Few Layer Graphene Nanoflake Chemoresistors for the Detection of Aromatic Volatile Organic Compounds. Sensors 2020, 20, 3413 10.3390/s20123413. PMC7349069 32560414

[ref19] The National Institute for Occupational Safety and Health (NIOSH). https://www.cdc.gov/niosh/npg/npgd0028.html (accessed 2021-07-02).

[ref20] Aneja V. P.; Schlesinger W. H.; Erisman J. W. Effects of agriculture upon the air quality and climate: Research, policy, and regulations. Environ. Sci. Technol. 2009, 43, 4234–4240. 10.1021/es8024403. 19603628

[ref21] Joshi A.; Gangal S. A.; Gupta S. K. Ammonia sensing properties of polypyrrole thin films at room temperature. Sens. Actuators, B 2011, 156, 938–942. 10.1016/j.snb.2011.03.009.

[ref22] Zhu S.; Sun H.; Liu X.; Zhuang J.; Zhao L. Room-Temperature NH_3_ sensing of graphene oxide film and its enhanced response on the laser-Textured silicon. Sci. Rep. 2017, 7, 147773 10.1038/s41598-017-15270-3. PMC5676790 29116161

[ref23] Wong Y. C.; Ang B. C.; Haseeb A. S. M. A.; Baharuddin A. A.; Wong Y. H. Review—Conducting Polymers as Chemiresistive Gas Sensing Materials: A Review. J. Electrochem. Soc. 2020, 167, 037503 10.1149/2.0032003JES.

[ref24] Benseddik E.; Makhlouki M.; Bernede J. C.; Lefrant S.; Proń A. XPS studies of environmental stability of polypyrrole-poly(vinyl alcohol) composites. Synth. Met. 1995, 72, 237–242. 10.1016/0379-6779(95)03285-1.

[ref25] Deshmukh K.; Basheer Ahamed M.; Deshmukh R. R.; Khadheer Pasha S. K.; Bhagat P. R.; Chidambaram K. Biopolymer Composites in Electronics; Elsevier Inc., 2017; pp 27–128.

[ref26] Li X. G.; Li A.; Huang M. R.; Liao Y.; Lu Y. G. Efficient and scalable synthesis of pure polypyrrole nanoparticles applicable for advanced nanocomposites and carbon nanoparticles. J. Phys. Chem. C 2010, 114, 19244–19255. 10.1021/jp107435b.

[ref27] Chartuprayoon N.; Hangarter C. M.; Rheem Y.; Jung H.; Myung N. V. Wafer-scale fabrication of single polypyrrole nanoribbon-based ammonia sensor. J. Phys. Chem. C 2010, 114, 11103–11108. 10.1021/jp102858w.

[ref28] Zhang X.; Manohar S. K. Bulk synthesis of polypyrrole nanofibers by a seeding approach. J. Am. Chem. Soc. 2004, 126, 12714–12715. 10.1021/ja046359v. 15469232

[ref29] Patois T.; Lakard B.; Martin N.; Fievet P. Effect of various parameters on the conductivity of free standing electrosynthesized polypyrrole films. Synth. Met. 2010, 160, 2180–2185. 10.1016/j.synthmet.2010.08.005.

[ref30] Yuan Y.; Lei A. Is electrosynthesis always green and advantageous compared to traditional methods?. Nat. Commun. 2020, 11, 802 10.1038/s41467-020-14322-z. 32029716 PMC7005282

[ref31] Tang X.; Lahem D.; Raskin J. P.; Gérard P.; Geng X.; André N.; Debliquy M. A Fast and Room-Temperature Operation Ammonia Sensor Based on Compound of Graphene with Polypyrrole. IEEE Sens. J. 2018, 18, 9088–9096. 10.1109/JSEN.2018.2869203.

[ref32] Zhang L.; Li C.; Liu A.; Shi G. Electrosynthesis of graphene oxide/polypyrene composite films and their applications for sensing organic vapors. J. Mater. Chem. 2012, 22, 8438–8443. 10.1039/c2jm16552j.

[ref33] Hong J. Y.; Yoon H.; Jang J. Kinetic study of the formation of polypyrrole nanoparticles in water-soluble polymer/metal cation systems: A light-scattering analysis. Small 2010, 6, 679–686. 10.1002/smll.200902231. 20127667

[ref34] Yang K.; Xu H.; Cheng L.; Sun C.; Wang J.; Liu Z. In vitro and in vivo near-infrared photothermal therapy of cancer using polypyrrole organic nanoparticles. Adv. Mater. 2012, 24, 5586–5592. 10.1002/adma.201202625. 22907876

[ref35] Wen J.; Tian Y.; Mei Z.; Wu W.; Tian Y. Synthesis of polypyrrole nanoparticles and their applications in electrically conductive adhesives for improving conductivity. RSC Adv. 2017, 7, 53219–53225. 10.1039/C7RA09725E.

[ref36] Olad A.; Shakoori S. Electromagnetic interference attenuation and shielding effect of quaternary Epoxy-PPy/Fe_3_O_4_-ZnO nanocomposite as a broad band microwave-absorber. J. Magn. Magn. Mater. 2018, 458, 335–345. 10.1016/j.jmmm.2018.03.050.

[ref37] García-Fernández M. J.; Buitrago-Sierra R.; Pastor-Blas M. M.; Soares O. S. G. P.; Pereira M. F. R.; Sepúlveda-Escribano A. Green synthesis of polypyrrole-supported metal catalysts: Application to nitrate removal in water. RSC Adv. 2015, 5, 32706–32713. 10.1039/C5RA03441H.

[ref38] Brédas J. L.; Scott J. C.; Yakushi K.; Street G. B. Polarons and bipolarons in polypyrrole: Evolution of the band structure and optical spectrum upon doing. Phys. Rev. B: Condens. Matter Mater. Phys. 1984, 30, 1023–1025. 10.1103/PhysRevB.30.1023.

[ref39] Le T.-H.; Kim Y.; Yoon H. Electrical and Electrochemical Properties of Conducting Polymers. Polymers (Basel, Switz.) 2017, 9, 150 10.3390/polym9040150. PMC6432010 30970829

[ref40] Balint R.; Cassidy N. J.; Cartmell S. H. Conductive polymers: Towards a smart biomaterial for tissue engineering. Acta Biomater. 2014, 10, 2341–2353. 10.1016/j.actbio.2014.02.015. 24556448

[ref41] Trchová M.; Stejskal J. Resonance Raman Spectroscopy of Conducting Polypyrrole Nanotubes: Disordered Surface versus Ordered Body. J. Phys. Chem. A 2018, 122, 9298–9306. 10.1021/acs.jpca.8b09794. 30418028

[ref42] Šetka M.; Calavia R.; Vojkůvka L.; Llobet E.; Drbohlavová J.; Vallejos S. Raman and XPS studies of ammonia sensitive polypyrrole nanorods and nanoparticles. Sci. Rep. 2019, 9, 9 10.1038/s41598-019-44900-1. 31186461 PMC6559985

[ref43] Lei J.; Cai Z.; Martin C. R. Effect of reagent concentrations used to synthesize polypyrrole on the chemical characteristics and optical and electronic properties of the resulting polymer. Synth. Met. 1992, 46, 53–69. 10.1016/0379-6779(92)90318-D.

[ref44] Pron A.; Rannou P. Processible conjugated polymers: From organic semiconductors to organic metals and superconductors. Prog. Polym. Sci. 2002, 27, 135–190. 10.1016/S0079-6700(01)00043-0.

[ref45] Sun J.; Shu X.; Tian Y.; Tong Z.; Bai S.; Luo R.; Li D.; Liu C. C. Facile preparation of polypyrrole-reduced graphene oxide hybrid for enhancing NH_3_ sensing at room temperature. Sens. Actuators, B 2017, 241, 658–664. 10.1016/j.snb.2016.10.047.

[ref46] Das M.; Roy S. Polypyrrole and associated hybrid nanocomposites as chemiresistive gas sensors: A comprehensive review. Mater. Sci. Semicond. Process. 2021, 121, 105332 10.1016/j.mssp.2020.105332.

[ref47] Tang X.; Raskin J. P.; Kryvutsa N.; Hermans S.; Slobodian O.; Nazarov A. N.; Debliquy M. An ammonia sensor composed of polypyrrole synthesized on reduced graphene oxide by electropolymerization. Sens. Actuators, B 2020, 305, 127423 10.1016/j.snb.2019.127423.

[ref48] Deokar G.; Casanova-Cháfer J.; Rajput N. S.; Aubry C.; Llobet E.; Jouiad M.; Costa P. M. F. J. Wafer-scale few-layer graphene growth on Cu/Ni films for gas sensing applications. Sens. Actuators, B 2020, 305, 127458 10.1016/j.snb.2019.127458.

[ref49] Šetka M.; Drbohlavová J.; Hubálek J. Nanostructured Polypyrrole-Based Ammonia and Volatile Organic Compound Sensors. Sensors 2017, 17, 562 10.3390/s17030562. PMC5375848 28287435

[ref50] Gustafsson G.; Lundström I.; Liedberg B.; Wu C. R.; Inganäs O.; Wennerström O. The interaction between ammonia and poly(pyrrole). Synth. Met. 1989, 31, 163–179. 10.1016/0379-6779(89)90812-6.

[ref51] Shen Y.; Yamazaki T.; Liu Z.; Meng D.; Kikuta T.; Nakatani N. Influence of effective surface area on gas sensing properties of WO_3_ sputtered thin films. Thin Solid Films 2009, 517, 2069–2072. 10.1016/j.tsf.2008.10.021.

[ref52] Katsnelson M. I.; Novoselov K. S. Graphene: New bridge between condensed matter physics and quantum electrodynamics. Solid State Commun. 2007, 143, 3–13. 10.1016/j.ssc.2007.02.043.

[ref53] Yang R.; Smyrl W. H.; Evans D. F.; Hendrickson W. A. Evolution of polypyrrole band structure: a scanning tunneling spectroscopy study. J. Phys. Chem. 1992, 96, 1428–1430. 10.1021/j100182a073.

[ref54] Camurlu P. Polypyrrole derivatives for electrochromic applications. RSC Adv. 2014, 4, 55832–55845. 10.1039/C4RA11827H.

[ref55] Bryan A. M.; Santino L. M.; Lu Y.; Acharya S.; D’Arcy J. M. Conducting Polymers for Pseudocapacitive Energy Storage. Chem. Mater. 2016, 28, 5989–5998. 10.1021/acs.chemmater.6b01762.

[ref56] Ahmed M. M.; Karimov K. S.; Moiz S. A. Temperature-dependent I-V characteristics of organic-inorganic heterojunction diodes. IEEE Trans. Electron Devices 2004, 51, 121–126. 10.1109/TED.2003.820650.

[ref57] Kangliang W.; Xiaoyan L.; Gang D.; Ruqi H. Simulation of carrier transport in heterostructures using the 2D self-consistent full-band ensemble Monte Carlo method. J. Semicond. 2010, 31, 084004 10.1088/1674-4926/31/8/084004.

[ref58] Farooqi B. A.; Ashraf A.; Farooq U.; Ayub K. Comparative study on sensing abilities of polyaniline and graphene polyaniline composite sensors toward methylamine and ammonia. Polym. Adv. Technol. 2020, 31, 3351–3360. 10.1002/pat.5058.

[ref59] Tang X.; Debliquy M.; Lahem D.; Yan Y.; Raskin J.-P. A Review on Functionalized Graphene Sensors for Detection of Ammonia. Sensors 2021, 21, 1443 10.3390/s21041443. 33669589 PMC7922188

[ref60] Joulazadeh M.; Navarchian A. H.; Niroomand M. A Comparative Study on Humidity Sensing Performances of Polyaniline and Polypyrrole Nanostructures. Adv. Polym. Technol. 2014, 33, 21461 10.1002/adv.21461.

[ref61] Mateos M.; Meunier-Prest R.; Suisse J. M.; Bouvet M. Modulation of the organic heterojunction behavior, from electrografting to enhanced sensing properties. Sens. Actuators, B 2019, 299, 126968 10.1016/j.snb.2019.126968.

[ref62] Tabačiarová J.; Mičušík M.; Fedorko P.; Omastová M. Study of polypyrrole aging by XPS, FTIR and conductivity measurements. Polym. Degrad. Stab. 2015, 120, 392–401. 10.1016/j.polymdegradstab.2015.07.021.

[ref63] Gazotti W. A.; Juliano V. F.; De Paoli M. A. Thermal and photochemical degradation of dodecylsulfate doped polypyrrole. Polym. Degrad. Stab. 1993, 42, 317–321. 10.1016/0141-3910(93)90227-A.

[ref64] Truong V. T. Thermal degradation of polypyrrole: effect of temperature and film thickness. Synth. Met. 1992, 52, 33–44. 10.1016/0379-6779(92)90017-D.

[ref65] Budrowski C.; Przyłuski J.; Kucharski Z.; Suwalski J. Stability of doped polypyrrole studied by Mössbauer spectroscopy. Synth. Met. 1990, 35, 151–154. 10.1016/0379-6779(90)90038-M.

[ref66] Casanova-Cháfer J.; García-Aboal R.; Atienzar P.; Llobet E. Gas Sensing Properties of Perovskite Decorated Graphene at Room Temperature. Sensors 2019, 19, 4563 10.3390/s19204563. PMC6832145 31635202

[ref67] Acik M.; Darling S. B. Graphene in perovskite solar cells: Device design, characterization and implementation. J. Mater. Chem. A 2016, 4, 6185–6235. 10.1039/C5TA09911K.

