# Supplementary material for: Graphene Loading with Polypyrrole Nanoparticles for Trace-Level Detection of Ammonia at Room Temperature
Source: ACS Appl Mater Interfaces. 2021 Aug 19;13(34):40909–21. doi: 10.1021/acsami.1c10559 (PMC8576760; doi:10.1021/acsami.1c10559)
Supplement: Supplementary file 1 — am1c10559_si_001.pdf [file am1c10559_si_001.pdf]

Supporting Information

Graphene loading with polypyrrole nanoparticles for trace-level detection of ammonia at room temperature

Juan Casanova-Chafer [1], Polona Umek [2], Selene Acosta[3], Carla Bittencourt [3] and Eduard Llobet [1]**

[1] Microsystems Nanotechnologies for Chemical Analysis (MINOS), Universitat Rovira i Virgili, 43007 Tarragona, Spain

[2] Jožef Stefan Institute, 10000 Ljubljana, Slovenia

[3] Chimie des Interactions Plasma–Surface (ChIPS), Research Institute for Materials Science and Engineering, Université de Mons, 7000 Mons, Belgium

AUTHOR INFORMATION

Corresponding authors

Juan Casanova-Chafer - Microsystems Nanotechnologies for Chemical Analysis (MINOS), Universitat Rovira i Virgili, 43007, Tarragona, Spain. Email: juan.casanova@urv.cat

Eduard Llobet - Microsystems Nanotechnologies for Chemical Analysis (MINOS), Universitat Rovira i Virgili, 43007, Tarragona, Spain. Email: eduard.llobet@urv.cat

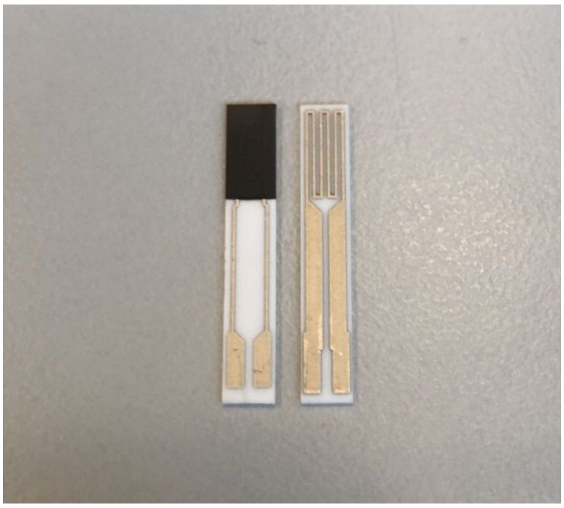

Figure S1. Layer of graphene nanoflakes loaded with PPy NPs onto alumina substrate.

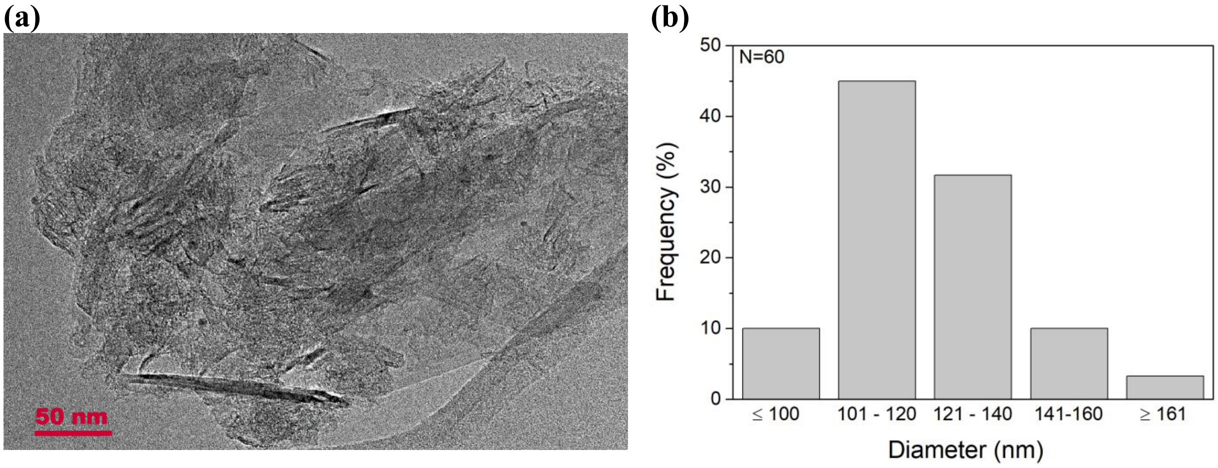

Figure S2. (**a**) HRTEM image at a lower magnification of the bare graphene. (**b**) PPy nanoparticle size distribution histogram. Above 75% of PPy NPs have diameter in the range of 101 to 140 nm.

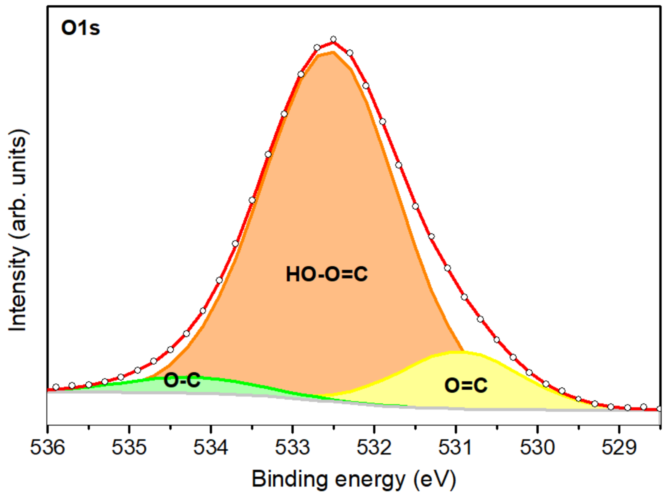

Figure S3. XPS O1s core level obtained for the bare PPy NPs.

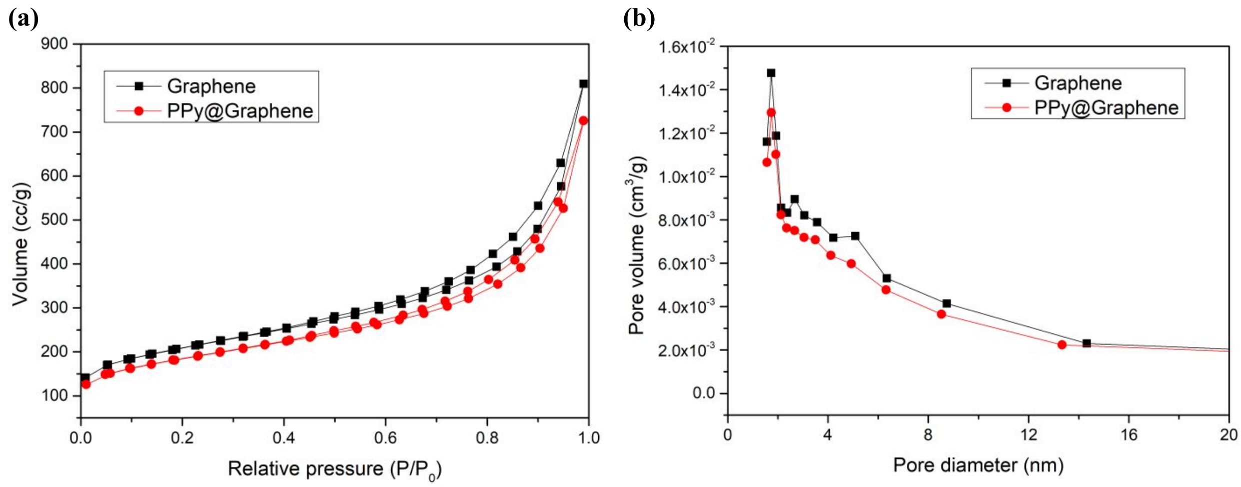

Figure S4. Adsorption-desorption isotherms for both samples (**a**). Pore diameter distribution for bare and PPy decorated graphene (**b**).

Table S1. Comparison of characterization results.

Sample	BET area (m^2/g)	Average pore diameter (nm)	Pore volume (cm^3/g)
**Graphene**	730	1.730	1.037
**PPy@Graphene**	644	1.735	0.993

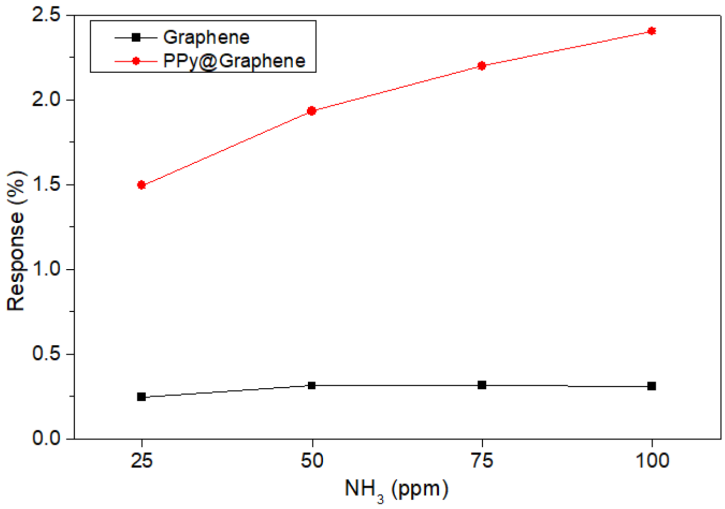

Figure S5. Calibration curves obtained for the detection of NH_3 (concentration range: 25-100 ppm).

(a) **(b)**

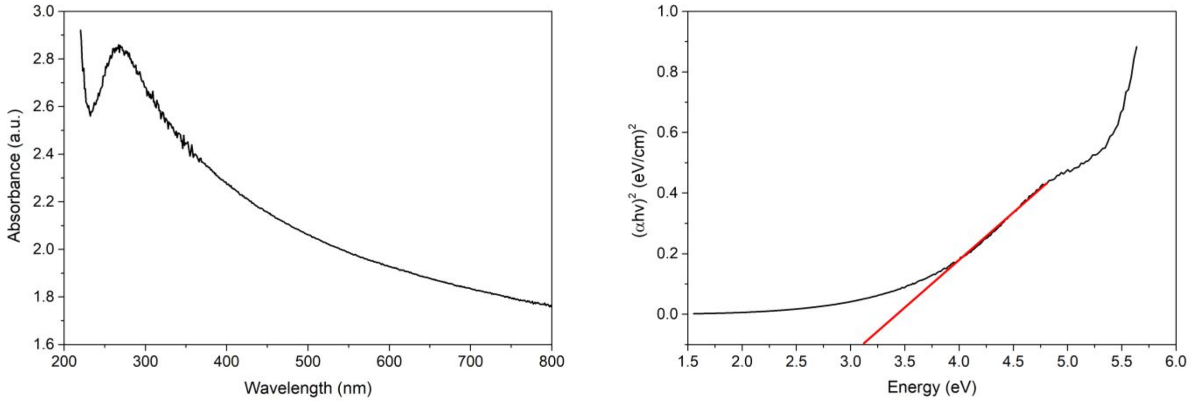

Figure S6. UV-Vis absorption spectrum for graphene nanoflakes (**a**) and Tauc plot for calculating their direct bandgap (**b**). Sample preparation comprised 5 mg of graphene added to 2 ml of ethanol in a 10 x 10 mm fluorescence quartz cuvette. Afterwards, the cuvette was sealed and subsequently deaerated by purging with an Ar gas stream for 10 minutes.

(a) **(b)**

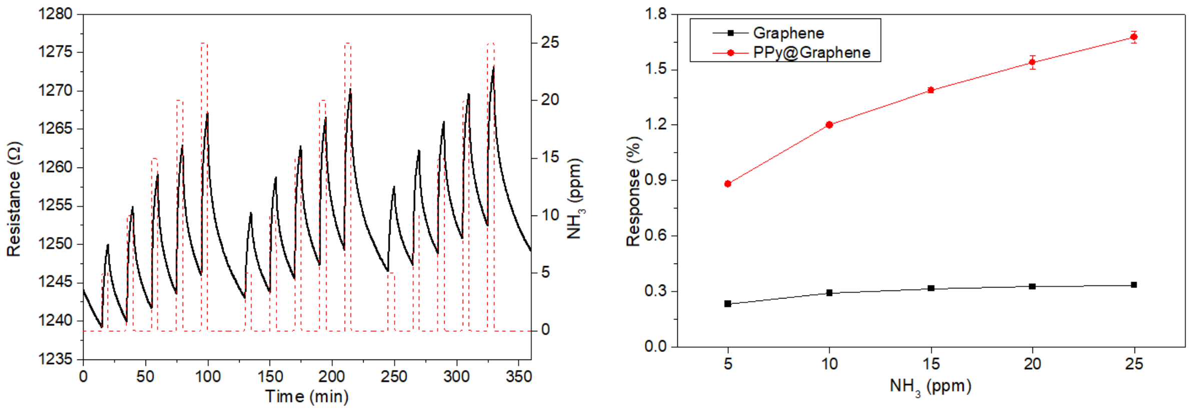

Figure S7. Example of electrical responses when detecting NH_3 at the concentration range of 5-25 ppm for the PPy@Graphene sensor (**a**). Comparison of the calibration curves obtained for bare and PPy loaded graphene (**b**). Higher responses and sensitivity can be observed for the PPy@Graphene sample.

(a) **(b)**

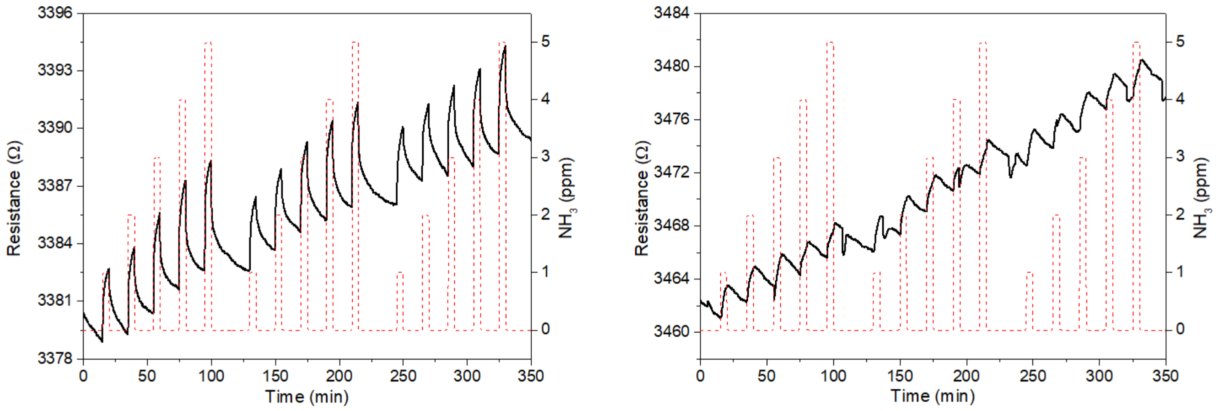

Figure S8. Examples of electrical responses when detecting low concentrations of NH_3 for the bare graphene sensor in dry (**a**) and humid (**b**) conditions.

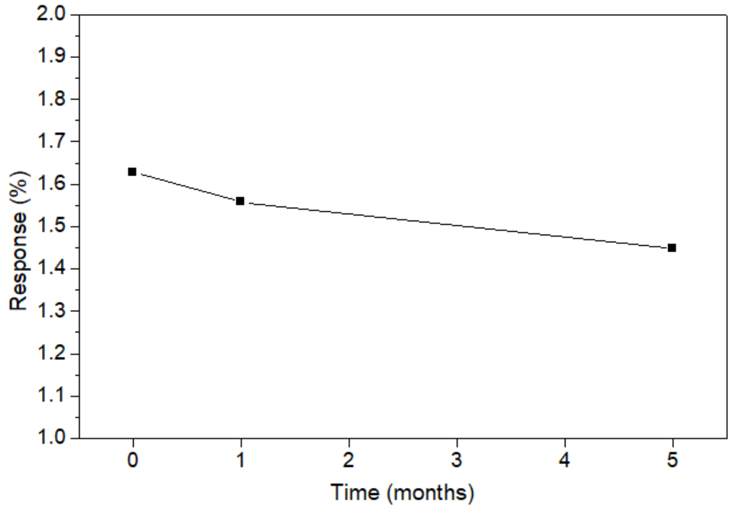

Figure S9. Stability study of the PPy@Graphene sensor, 50 ppm of NH_3 were measured over a 5-month period. A slightly higher slope during the first month can be observed. Nonetheless, during the following months, the slope was lowered, revealing a high sensor stability in the long term.

Table S2. Relative area (%A) of the different components obtained from the C 1s core level peak. The comparison was done with the freshly synthesized sample before the gas sensing measurements and after 5-months of use for detecting NH_3.

Component	%A Before	%A After
**sp^2**	45.1	42.4
**sp^3**	34.9	36.9
**C-O/C-N**	11.4	11.7
**C=O/C=N**	5.8	6.5
**O-C=O/N-C=O**	2.8	2.5

Table S3. Relative area (%A) of the different components obtained from the N 1s core level peak. The comparison was done with the freshly synthesized sample before the gas sensing measurements and after 5-months of use for detecting NH_3.

Component	%A Before	%A After
**NH**	54	40
**C=N**	9	27
**$C-N^+$**	32.1	12.3
**$C=N^+$**	4.9	12.1
**Amines**	-	7.7

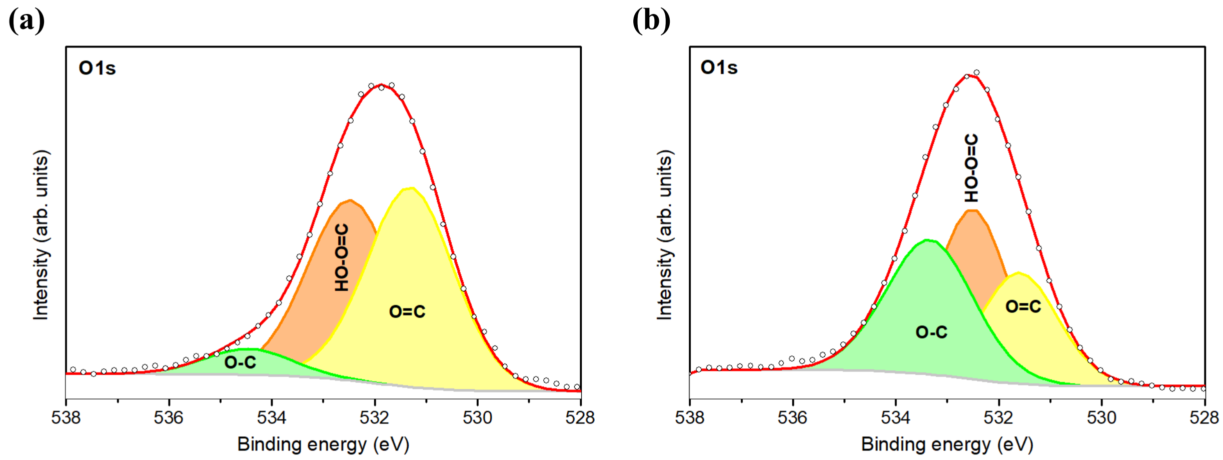

Figure S10. XPS O 1s core level obtained for the sample PPy@Graphene before (**a**) and after (**b**) NH_3 gas sensing.

Table S4. Comparison of the NH_3 sensing performance for polypyrrole (PPy)/graphene hybrids operated at room temperature. Sensitivity coefficients calculated as the response (%) / concentration applied. LOD: limit of detection. NA: not available data. *Color code*: Green and Red are for positive and negative features, respectively. Orange is not positive nor negative.

Nanomaterial	Polymerization procedure	Ease of synthesis	Solvent-free	Sensitivity Coefficient	Flow rate (sccm)	Carrier gas	LOD (ppb)	Stability test	Moisture cross-sensitivity test	Repeatability test	Reference
PPy NPs – Graphene	Chemical	Yes	Yes	0.88	100	Air	419	Yes	Yes	Yes	This work
PPy - GO	Chemical	Yes	No	0.029	NA	Air	NA	No	No	No	[1]
Thin PPy layer - Graphene	Electrochemical	No	No	1.7	1000	Air	NA	Yes	Yes	Yes	[2]
PPy – Single Layer Graphene	Electrochemical	No	No	7.5	NA	Air	0.04	No	No	Yes	[3]
PPy - rGO	Chemical	Yes	Yes	0.37	NA	Air/N_2	NA	No	No	No	[4]
TiO_2 NPs – Ppy - rGO	Chemical	Yes	No	2.04	NA	N_2	1000	Yes	Yes	Yes	[5]
PPy film - GO	Electrochemical	Yes	No	0.1	1000	N_2	NA	No	No	No	[6]

References

[1] Patil, P., Gaikwad, G., Patil, D. R., Naik, J., Gas Sensitivity Study of Polypyrrole Decorated Graphene Oxide Thick Film. *J. Inst. Eng. Ser. D* 2016, 97, 47–53.

[2] Tang, X., Lahem, D., Raskin, J. P., Gérard, P., Geng, X., André, N., Debliquy, M., A Fast and Room-Temperature Operation Ammonia Sensor Based on Compound of Graphene with Polypyrrole. *IEEE Sens. J.* 2018, 18, 9088–9096.

[3] Yoon, T., Jun, J., Kim, D. Y., Pourasad, S., Shin, T. J., Yu, S. U., Na, W., Jang, J., Kim, K. S., An ultra-sensitive, flexible and transparent gas detection film based on well-ordered flat polypyrrole on single-layered graphene. *J. Mater. Chem. A* 2018, 6, 2257–2263.

[4] Tiwari, D. C., Atri, P., Sharma, R., Sensitive detection of ammonia by reduced graphene oxide/polypyrrole nanocomposites. *Synth. Met.* 2015, 203, 228–234.

[5] Xiang, C., Jiang, D., Zou, Y., Chu, H., Qiu, S., Zhang, H., Xu, F., Sun, L., Zheng, L., Ammonia sensor based on polypyrrole-graphene nanocomposite decorated with titania nanoparticles. *Ceram. Int.* 2015, 41, 6432–6438.

[6] Zhang, L., Li, C., Liu, A., Shi, G., Electrosynthesis of graphene oxide/polypyrene composite films and their applications for sensing organic vapors. *J. Mater. Chem.* 2012, 22, 8438–8443.
